# Extraction and LC-MS/MS Analysis of Ciguatoxins: A Semi-Targeted Approach Designed for Fish of Unknown Origin

**DOI:** 10.3390/toxins13090630

**Published:** 2021-09-08

**Authors:** Astrid Spielmeyer, Christopher R. Loeffler, Dorina Bodi

**Affiliations:** 1 Department Safety in the Food Chain, German Federal Institute for Risk Assessment, National Reference Laboratory for Monitoring of Marine Biotoxins, Max-Dohrn-Str. 8-10, 10589 Berlin, Germany; Christopher.Loeffler@bfr.bund.de (C.R.L.); Dorina.Bodi@bfr.bund.de (D.B.); 2 Department of Pharmacy, School of Medicine and Surgery, University of Napoli Federico II, Via D. Montesano 49, 80131 Napoli, Italy

**Keywords:** chromatography, ciguatoxins, food safety, food contaminants, mass spectrometry, N2a, validation

## Abstract

Ciguatoxins (CTXs) are polyether marine biotoxins that can cause ciguatera poisoning (CP) after the consumption of fish or invertebrates containing sub ppb levels; concentrations that present a challenge for current extraction and analysis methods. Here, a newly developed and (partly) validated single-day extraction protocol is presented. First, the fish sample is broken-down by enzymatic digestion, followed by extraction and extract clean-up by defatting and two solid-phase extractions. Final extracts were investigated using two different CTX-analysis methods; an in vitro cytotoxicity assay (N2a-assay) and by LC-MS/MS. Validation was performed for both fillet and freeze-dried samples of snapper, parrotfish, and grouper spiked with CTX1B, 52*-epi-*54-deoxyCTX1B, 54-deoxyCTX1B, and CTX3C. Based on recovery rates (35–88%) and matrix effects (66–116%) determined by LC-MS/MS, the enzyme protocol is applicable to various matrices. The protocol was applied to naturally contaminated fish tissue (*Lutjanus bohar*) obtained during a CP incident in Germany. Several potential CTX congeners were identified by a two-tier LC-MS/MS approach (screening of sodium adducts, high-resolution or low-resolution confirmation via ammonium adducts). Inclusion of >30 known CTX congeners into the LC-MS/MS methods and single-day sample preparation make the method suitable for analysis of ciguatera suspect samples at sub ppb levels also with undisclosed CTX profiles.

## 1. Introduction

**Figure 1:**
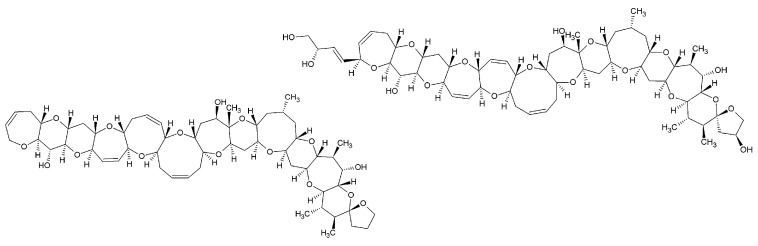
Structures of the commercially available CTX congeners CTX3C (**left**) and CTX1B (**right**; stereochemistry taken from [7]).

Ciguatoxins (CTXs) are marine biotoxins produced by dinoflagellates in the genera *Gambierdiscus* and *Fukuyoa* [1,2,3,4]. CTXs are lipophilic polyether molecules (Figure 1) with (computed) logP values of, e.g., 2.5 for CTX1B [5] and 4.7 for CTX3C [6]. They accumulate within the food web and undergo biotransformation processes leading to a variety of compounds. Currently, over 30 different CTX congeners (including epimers) are known, differing in the number of condensed rings and in the presence/absence of side chains [7]. Based on their skeletal structure and the first region of isolation, four groups of CTXs are differentiated, namely CTX3C, CTX4A (both Pacific CTX and P-CTX), Caribbean CTX (C-CTX), and Indian Ocean CTX (I-CTX; Table 1). The structural elucidation of I-CTXs has not been accomplished so far [8,9,10].

CTXs can result in ciguatera poisoning (CP), the most commonly reported non-bacterial illness related to seafood (a review about incident rates, symptoms is provided by, e.g., [11]). CTXs are highly potent, capable of causing symptoms after the consumption of fish or invertebrates containing levels in the sub ppb range. The US Food and Drug Administration (FDA) set guidance values of 0.01 µg kg^−1^ for CTX1B equivalents and 0.1 µg kg^−1^ for C-CTX-1 equivalents in finfish ([12], based on [13]).

**Table 1:** Formulas and (high-resolution) *m*/*z* of precursor and product ions of ciguatoxins (CTX) congeners considered in this study.

CTX Congener	Formula	[M + H − 3H_2_O]^+^	[M + H − 2H_2_O]^+^	[M + H − H_2_O]^+^	[M + H]^+^	[M + NH_4_]^+^	[M + Na]^+^
**CTX4A group ^1^**							
CTX4A/B	C_60_H_84_O_16_	1007.55152	1025.56208	1043.57265	1061.58321	1078.60976	1083.56516
M-*seco*-CTX4A/B	C_60_H_86_O_17_	1025.56208	1043.57265	1061.58321	1079.59378	1096.62033	1101.57572
52*-epi-*54-deoxyCTX1B 54-deoxyCTX1B	C_60_H_86_O_18_	1041.55700	1059.56756	1077.57813	1095.58869	1112.61524	1117.57064
CTX1B 52-/54*-epi*CTX1B 52*-epi-*54*-epi*CTX1B 54-deoxy-50-hydroxyCTX1B	C_60_H_86_O_19_	1057.55191	1075.56248	1093.57304	1111.58361	1128.61016	1133.56555
7-oxoCTX1B	C_60_H_86_O_20_	1073.54683	1091.55739	1109.56796	1127.57852	1144.60507	1149.56047
7-hydroxyCTX1B	C_60_H_88_O_20_	1075.56248	1093.57304	1111.58361	1129.59417	1146.62072	1151.57612
4-hydroxy-7-oxoCTX1B	C_60_H_88_O_21_	1091.55739	1109.56796	1127.57852	1145.58909	1162.61564	1167.57103
**CTX3C group ^1^**							
CTX3C/B	C_57_H_82_O_16_	969.53587	987.54643	1005.55700	1023.56756	1040.59411	1045.54951
51-hydroxyCTX3C	C_57_H_82_O_17_	985.53078	1003.54135	1021.55191	1039.56248	1056.58903	1061.54442
M-*seco*-CTX3C 2-hydroxyCTX3C	C_57_H_84_O_17_	987.54643	1005.55700	1023.56756	1041.57813	1058.60468	1063.56007
M-*seco*-CTX3C methyl acetal	C_58_H_86_O_17_	1001.56208	1019.57265	1037.58321	1055.59378	1072.62033	1077.57572
51-hydroxy-2-oxoCTX3C	C_57_H_82_O_18_	1001.52570	1019.53626	1037.54683	1055.55739	1072.58394	1077.53934
2,3-dihydroxyCTX3C	C_57_H_84_O_18_	1003.54135	1021.55191	1039.56248	1057.57304	1074.59959	1079.55499
A-*seco*-51-hydroxyCTX3C	C_57_H_86_O_18_	1005.55700	1023.56756	1041.57813	1059.58869	1076.61524	1081.57064
2,3,51-trihydroxyCTX3C	C_57_H_84_O_19_	1019.53626	1037.54683	1055.55739	1073.56796	1090.59451	1095.54990
**C-CTX group ^2^**							
C-CTX-1/2	C_62_H_92_O_19_	1087.59886	1105.60943	1123.61999	1141.63056	1158.65711	1163.61250
C-CTX-3/4	C_62_H_94_O_19_	1089.61451	1107.62508	1125.63564	1143.64621	1160.67276	1165.62815
C-CTX reaction product 8	C_61_H_88_O_18_	1055.57265	1073.58321	1091.59378	1109.60434	1126.63089	1131.58629
C-CTX reaction product 9	C_61_H_90_O_18_	1057.58830	1075.59886	1093.60943	1111.61999	1128.64654	1133.60194
C-CTX-1127	C_61_H_90_O_19_ *	1073.6	1091.6	1109.6	1127.6	1144.6	1149.6
C-CTX-1157	C_62_H_92_O_20_ *	1103.6	1121.6	1139.6	1157.6	1174.6	1179.6
**I-CTX group ^3^**							
I-CTX-1/2	C_62_H_92_O_19_	1087.59886	1105.60943	1123.61999	1141.63056	1158.65711	1163.61250
I-CTX-3/4	C_62_H_92_O_20_	1103.59378	1121.60434	1139.61491	1157.62547	1174.65202	1179.60742
I-CTX-5	C_62_H_90_O_19_	1085.58321	1103.59378	1121.60434	1139.61491	1156.64146	1161.59685
I-CTX-6	C_62_H_90_O_20_	1101.57813	1119.58869	1137.59926	1155.60982	1172.63637	1177.59177

*—formula unknown/not confirmed, information of a (potential) formula of congeners by ^1^ [14], ^2^ [15,16,17], ^3^ [8].

Within the European Union, food contaminants like marine biotoxins are regulated both in terms of maximum levels (Regulation (EC) 853/2004, [18]) and recognized analytical methods (Commission Implementing Regulation (EU) 2019/627, [19]). Although it is stated in Regulation (EC) 853/2004 that CTX-containing “fishery products […] must not be placed on the market”, currently, there is no ‘recognized method’ regarding CTX analysis (according to Commission Implementing Regulation (EU) 2019/627). Full method validation, which is necessary for its implementation in the legislation, is hampered by the lack of (sufficient) analytical standards and (certified, commercially obtainable) reference material. To date (August 2021), only two CTX congeners (CTX1B, CTX3C) are available for purchase. Therefore, all other congeners, for which there are >30, must be isolated and purified from naturally contaminated material.

Because of these analytical limitations, a two-tier approach for CTXs is typically performed (described in [13]). Initially, a sample extract is investigated via a screening test utilizing a mouse (*Mus musculus*) neuroblastoma cell line (Neuro-2a, N2a) in a cytotoxicity assay (N2a-assay). The sensitivity and specificity of the N2a-assay enable a semi-quantitative estimation of the total effect of all toxins present in the sample, based on CTXs’ mode of action on the voltage-gated sodium channel (Na_v_). When compared to a known standard solution, an extract of a defined sample amount (e.g., 5.00 g) can be functionally described in standard equivalents (e.g., µg of CTX3C equivalents per kg of sample tissue). Following the screening type assay, tier two involves a qualitative confirmation of the respective toxins via LC-MS/MS analysis [13].

Due to their lipophilic nature and high potency at low concentrations, CTX analyses in complex matrices (e.g., fish tissue) are challenging. Sample preparation protocols, therefore, involve several steps, consisting of extraction, protein precipitation, liquid-liquid partitioning, e.g., for defatting, drying/evaporation steps, and one or more solid phase extraction(s) (SPE; reviews, e.g., provided by [20,21]). While effective, these protocols have several drawbacks, including the required time (e.g., due to overnight precipitation, drying steps of aqueous phases [22,23,24]) and the usage of larger solvent volumes (e.g., 19 mL [23] or 6 mL [24,25] per gram tissue for initial extraction), where large solvent volumes can create equally large chemical waste disposal volumes and can be difficult to handle.

Most sample preparation methods include a mechanical treatment of the fish fillet for matrix break-up and homogenization combined with the extraction by acetone or (aqueous) methanol. However, CTXs can be associated with proteins [26,27,28]; therefore, a solely mechanical treatment may result in an incomplete extraction of CTXs from the tissue. To overcome this limitation, a new approach was tested in this study, based on enzymatic hydrolysis of the protein matrix using papain. Papain was previously described to be suitable for fish protein hydrolysis [29]. The digestion was followed by extraction, defatting, and SPE. By minimizing evaporation steps and avoiding the evaporation of aqueous phases, the whole sample preparation can be performed by one person within one working day (7–8 h for 4–6 samples). Extracts were tested for their applicability for analysis both in the N2a-assay and LC-MS/MS to provide one extraction protocol suitable for both analytical approaches.

In general, sample preparation methods for CTX analysis are often time-consuming due to several clean-up steps needed for LC-MS/MS analyses [23,25,30]. Fast extraction methods have been developed, but these were typically designed with a focus on a specific congener (CTX1B in case of [31]) or only (medium) polar congeners with chromatograms displaying a pronounced increase in the baseline with increasing run time [32]. Therefore, while suitable for their specifically designed task, their utilization in a broader investigation could inhibit the detection of unpolar congeners such as CTX3C. Thus, the aim of this study was to develop a protocol that can reduce the time required to one working day, which is also suitable for a broad range of CTX congeners. In addition to saving time, the protocol’s ease of use was considered as well as minimizing the solvent volumes compared to current methods in order to avoid the usage of (large) separation funnels. Here, the new enzyme protocol was developed with the intent of utilizing small volume reusable glass centrifuge tubes throughout the preparation process for easier handling.

LC-MS/MS methods reported in the literature often focus on specific congeners, e.g., only (selected) C-CTX or P-CTX [23,24,25,31,32]. This specific focus, while helpful for constraining efforts when materials or methods were limited, can lead to a false-negative result as other known congeners might be overlooked. Furthermore, the reliance on *a priori* assumptions for region-specific CTX groups, based on a product’s presumed region of capture, can lead to false-negative results, e.g., in situations of species substitution or in a scenario where a species (CTX-producing or CP-vector) is introduced to a novel or previously undescribed region [33]. Therefore, to avoid this potential error, the LC-MS/MS method presented here includes >30 congeners reported in the literature (Table 1). It is considered as a semi-targeted workflow, as reference standards are lacking for most compounds to provide confirmative information such as retention time or fragmentation pattern [34]. Because this congener approach is inclusive of the known spectrum of CTXs, it is applicable for both *a priori* assumed CTX groups as well as blind, unbiased sample analysis.

The availability of standards and naturally contaminated materials represents a critical bottleneck, which restrict a laboratory’s ability to gain the experience necessary for providing CTX analysis. Because these materials are severely limited, an exchange of available research material among laboratories, with the intent of developing and harmonizing existing methods and training, is needed until sufficient standard material is widely available [33]. However, the material exchange can be costly and logistically prohibitive. Therefore, to resolve several transfer issues drying of sample material can lower shipping costs (reduced weight, no insulation/ice required, ensuring a temperature and biologically stable product without the need for expedited shipping). Accordingly, the method described herein was developed for (freeze-)dried fish tissue but is also applicable for raw/frozen tissue, allowing for a range of sample conditions and material transfer capabilities. Validation was performed for three different fish genera (*Lutjanus*, *Scarus*, *Epinephelus*) to cover a broad range of potential CP-associated fish types and sample matrices.

## 2. Results and Discussion

### 2.1. Extraction Protocol Development

The enzyme protocol presented here includes a first extraction step with acetone for CTX extraction from the matrix. Most methods utilize acetone or aqueous methanol for extraction (reviews by [20,21]). However, both solvents are mixable with water leading to a transition of water and water-soluble matrix constituents from the fish sample into the extract. Due to the enzymatic treatment of the fish tissue, the hydrolyzed sample was expected to contain more polar protein fragments that can be extracted by polar solvents than solely mechanical treated samples. Therefore, the initial extraction was modified. The subsequent addition of saturated sodium chloride increases the polarity of the aqueous phase. Consequently, acetone blends with ethyl acetate that is not mixable with water in the last step. This way, both the water content and the matrix load of the extract were remarkably reduced. Additional extraction steps from the fish matrix were not conducted as solvents led to denaturation and strong agglomeration of the solid particles. Thus, more extraction steps were considered less effective, an observation further supported by the results of other studies [23]. The raw extract was washed with saturated sodium chloride to remove excess water and polar matrix components from the organic phase.

The defatting procedure was taken from [23] and modified using smaller solvent volumes. It involves the addition of saturated sodium carbonate and 5% citric acid and allows basic and acidic matrix constituents to be removed by *n*-hexane extraction. As a higher sample weight (5 g compared to 2 g by [23]) and lower *n*-hexane volumes were utilized, an additional defatting step was included prior to the addition of base and acid to the aqueous methanol phase.

To enable a time-efficient sample preparation, the reversed-phase (RP) SPE was conducted first, followed by a normal phase (NP) SPE (see also [31]). Some sample clean-up procedures include an NP SPE after the defatting step [23,25]. This requires a reduction of the aqueous methanol to dryness to reconstitute the sample in a solvent suitable for NP SPE. In contrast to that, the defatted sample could be applied directly to an RP SPE. With the material utilized here, no elution of CTXs was observed with 80 vol% methanol, and no adjustment of the water content was necessary. Elution was realized with two different solvents as ethyl acetate and water are not miscible. Acetonitrile was chosen to remove the remaining water from the column material before acidified ethyl acetate was applied. The addition of acetic acid proved necessary, as without acidification recovery rates below 25% were obtained for CTX1B (20%), 52*-epi-*54-deoxyCTX1B (21%), and 54-deoxyCTX1B (23%). The impact on CTX3C was less pronounced (55%, all data refer to snapper fillet). The RP SPE material utilized contains a weak anion exchanger. Apparently, acidic conditions were necessary to overcome the interaction of the three CTX4A-group congeners with the sorbent material. This implies an influence of the but-3-ene-1,2-diol side chain at ring A on the retention of these congeners on the SPE phase used here.

The volume of the RP SPE eluate needs to be reduced before dilution with *n*-hexane to apply a small volume to the NP SPE (here: 4 mL). Application of larger volumes led to a full transfer of CTX3C into the filtrate, but other congeners such as 52*-epi-*54-deoxyCTX1B or 54-deoxyCTX1B were (partly) found in that fraction as well (NP SPE delivers two fractions, “filtrate” and “eluate”, see Section 4.2 and Section 4.2.4 for details). This observation shows that, when developing methods for CTXs, it is important to include more than one CTX whenever possible. The focus on only one congener can result in potentially inaccurate conclusions regarding the optimal (SPE) conditions, at least in the case where the analysis of multiple CTXs is required.

**Figure A1:**
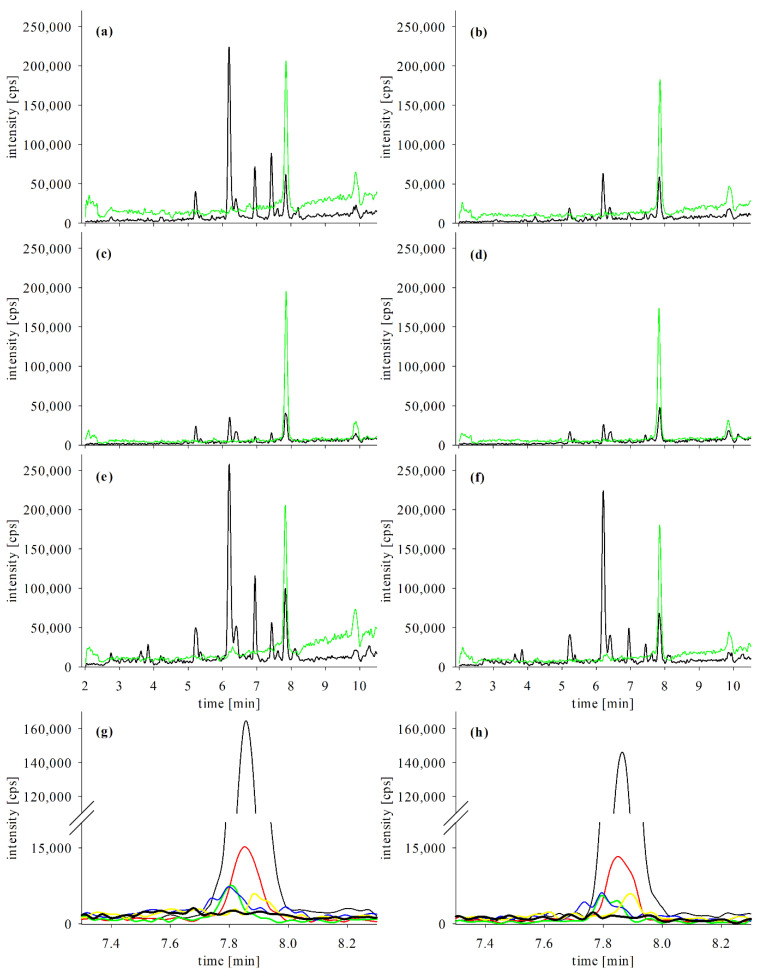
(**a**–**f**) Total Ion Count chromatograms of blank matrix eluate (green) and filtrate (black) extracts of (**a**,**b**) snapper, (**c**,**d**) parrotfish, and (**e**,**f**) grouper with left and right columns showing fillet and freeze-dried samples, respectively; analyses were performed for sodium adducts according to Section 4.3.1; (**g**,**h**) Extracted Ion Chromatograms of the congeners causing the peak at 7.85 min in all extracts with M-*seco*-CTX4A/B (black), (52*-epi-*)54-deoxyCTX1B (red), CTX3C (blue), I-CTX-5 (green), and 51-hydroxyCTX3C (yellow) in snapper eluate of (**g**) fillet and (**h**) freeze-dried material; for the congener 2-hydroxyCTX3C (black bold line), no peak was observed at that retention time.

Dilution of the reduced RP SPE eluate with *n*-hexane and the addition of acetic acid to the ethyl acetate enabled a separation of CTX congeners and matrix compounds between eluate and filtrate during NP SPE. CTX1B, 52-*epi*-54-deoxyCTX1B, and 54-deoxyCTX1B were transferred into the eluate, while the majority of matrix components passed into the filtrate (Figure A1). This facilitated the evaluation of potential CTXs that have no readily available standards. Eluates of blank samples showed almost no peaks for all congeners investigated (Figure A1), so signals observed in this fraction are likely to derive from CTX congeners instead of matrix components.

**Figure A2:**
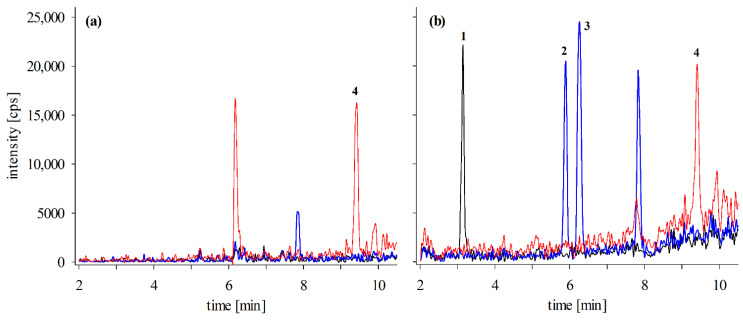
Extracted Ion Chromatograms of an extract of snapper fillet fortified prior to sample preparation with 0.2 µg kg^−1^ CTX1B (1, black line), 52*-epi-*54-deoxyCTX1B (2, blue line), 54-deoxyCTX1B (3, blue line), and 0.4 µg kg^−1^ CTX3C (4, red line) with (**a**) filtrate and (**b**) eluate fraction; *m*/*z* are provided in Table 1; sample preparation and analysis were performed according to Section 4.2 (respective Section 4.4.1; extract utilized for determination of recovery rate) and Section 4.3.1.

CTX3C was found to split up between eluate and filtrate (Figure A2) at a ratio of 2:3, irrespective of the matrix. No further attempts were conducted to enable a full transfer of CTX3C into the eluate fraction, as the fractionation of matrix components and CTX congeners was considered more relevant. Nevertheless, CTX3C can be investigated with this setup as well, and LODs and LOQs are comparable to the congeners detected in the eluate only (see Section 2.2.2). Currently, there is no specific guidance with respect to CTX3C contents beyond the EU regulation [18,19].

Within the enzyme protocol, acetic acid was added with a total amount of 0.1 vol% to ethyl acetate. Acetic acid was preferred over formic acid, as C-CTX-1 has been shown to undergo a transformation (methylation) when incubated under strong acidic conditions with 9% hydrochloric acid (HCl) or 9% formic acid in methanol or acetonitrile. The reactions were more pronounced with HCl [35]. In order to avoid artificial methylation of CTXs during sample preparation, acetic acid was utilized as it is a weaker acid (pKa 4.75, [36]) than formic acid (pKa 3.75, [37]) or HCl (computational pKa −5.86, [38]). Acidic conditions favor the epimerization of CTX congeners. This fact can be utilized for structure elucidation [14] or standard preparation [30]. For full conversion, strongly acidic conditions with HCl (0.1 M) and elevated temperatures (55 °C) were employed [30]. Due to solvent evaporation during sample preparation, a concentration of acetic acid can occur, which might favor epimerization. However, extraction efficiencies of 52*-epi-*54-deoxyCTX1B and 54-deoxyCTX1B were comparable (see Section 2.2.1); thus, epimerization was not expected to be enhanced during the evaporation steps. In that case, extraction efficiencies should be higher for 52*-epi-*54-deoxyCTX1B. Acetic acid was considered to have no influence on the CTX recovery and stability within the sample preparation described here; however, prolonged storage (e.g., overnight) of acidified solutions might favor epimerization. Thus it is recommended to conduct sample preparation within one day as soon as acidic conditions are employed.

### 2.2. Method Validation

#### 2.2.1. Recovery Rates, Matrix Effects, Extraction Efficiency, and Sample Stability

Extraction efficiencies were comparable for all representative fish tissue matrices (species) as well as the tissue condition of fillet and freeze-dried materials (Table 2). Therefore, the method can be considered suitable for a broad range of matrices. Four CTX congeners of different polarities and groups were investigated. Similar extraction efficiencies were obtained with slightly higher values for the least polar compound CTX3C (Table 2). Using the enzyme protocol, the full range of CTX congeners can be covered without major discrimination of high and low oxidized compounds.

The recovery rates ranged from 35 to 88% for all analytes and matrices (only the sum of CTX3C was taken into account; Table 2). Stewart et al. (2010) and Wu et al. (2011) conducted spiking experiments with several fish species and found recoveries from 27 to 85% for CTX1B [32,39]. For the same congener, values from 4.5 to 85.1% were reported in several fillet samples of *Scomberomorus commerson* (Spanish mackerel, [40]). In the case of CTX1B and CTX3C, recoveries between 86 and 107% were obtained for *Seriola quinqueradiata* (amberjack) and *Pagrus major* (seabream) [23]. This brief overview shows that recovery rates in CTX analyses cover the entire range and depend not only on the protocol utilized but also on the fish species. Method development studies should, therefore, include more than one species, covering, e.g., different fat contents, to demonstrate the applicability to different matrices, a point of importance previously discussed for the N2a-assay [41].

According to the Standard Method Performance Requirements of the AOAC (Appendix F, Table A5 in that document), recovery rates should be between 40 and 120% for an analyte content of 1 ppb [42]. This point is fulfilled by all analytes and samples except CTX1B, 52-epi-54-deoxyCTX1B, and 54-deoxyCTX1B in grouper fillet. According to the guidance document SANTE/12682/2019, recovery rates should range between 70 and 120%; however, values between 30 and 140% can be accepted if the repeatability is below 20% (for this parameter, see Section 2.2.4) and if the reasons for lower/higher recovery rates are known [43]. Extraction efficiencies were comparable for all matrices; therefore, low recovery rates for grouper fillet are ascribed to a higher signal suppression during LC-MS/MS analysis. Consequently, no further adjustments for the sample preparation protocol were conducted, and values of 35 to 39% were accepted.

For the grouper fillet, a liquid (fat) residue was obtained after removing the solvent of the raw extract. Due to several defatting steps, the impact of fat itself on the recovery rate is not expected. Fat-soluble constituents that were not removable during extract clean-up probably led to the observed signal suppression (Table 2). Based on a dry weight of 20% (determined for fillet without skin and bones, see Section 4.1), 0.5 g freeze-dried material corresponds to 2.5 g fillet. Extracts of freeze-dried grouper showed less signal suppression than the corresponding fillet sample, and the values were comparable to snapper samples (Table 2), probably due to the lower matrix load. If samples with high-fat content are investigated (e.g., viscera), a reduction of the initial sample weight might be appropriate to reduce matrix suppression in LC-MS/MS analysis.

Matrix effects are not always a factorial component investigated in method validation studies, although they are often a suspected reason for recovery rates falling below expectations. On day 0 of the validation experiments, matrix effects between 66 and 116% were found (Table 2), corresponding to a maximal signal suppression of 34%. This is comparable to values reported in other studies (22–40%), although signal suppressions up to 75% are reported as well [25,31,39,44]. Fat-soluble constituents are often considered as a primary reason for the observed matrix effects [31,39]. This is underlined by the observation that the highest signal suppression was observed for the comparable fatty grouper matrix. Nagae et al. (2021) reported mean recovery rates of 87% to 107% for CTX1B and CTX3C in snapper and grouper [23]. Although matrix effects were not determined separately in that study, the results imply a low impact of the matrix on the LC-MS/MS analysis. One reason for the lower matrix effects might be the higher solvent volume utilized during sample preparation which might be more effective for the removal of co-eluting matrix constituents. Another potential reason could be the usage of less sample matrix (2 g compared to 5 g in this study).

**Figure 2:**
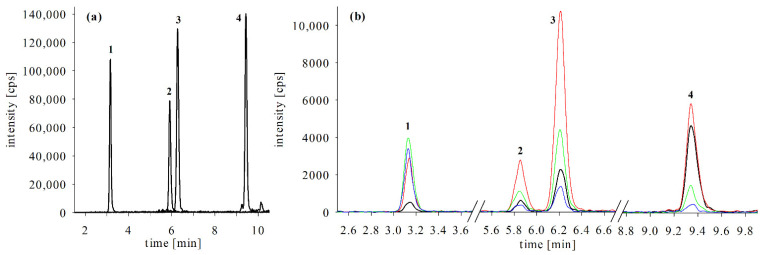
Extracted ion chromatograms of a CTX standard in methanol with 4 µg L^−1^ CTX1B (1), 52*-epi-*54-deoxyCTX1B (2), 54-deoxyCTX1B (3), and 8 µg L^−1^ CTX3C (4) with (**a**) detection of sodium adducts [M + Na]^+^ (method includes 20 ion transitions with 50 ms dwell time each) and (**b**) low-resolution detection of the product ions of ammonium adducts with [M + H]^+^ (black), [M + H − H_2_O]^+^ (red), [M + H − 2H_2_O]^+^ (green), [M + H − 3H_2_O]^+^ (blue); *m*/*z* are provided in Table 1; for ammonium adducts, analysis was performed in scheduled MRM mode with a 300 ms scan time per transition.

In contrast to snapper and grouper, the eluate of parrotfish samples showed almost no signal suppression; rather, the signal for CTX3C showed enhancement in eluate and filtrate. These observations underline a major problem for CTX quantitation when investigating and comparing several matrices. When different fish species are compared concerning their CTX profile and contents, quantitative differences might (partially) be the result of diverse matrix effects. This shortcoming could be overcome by the workflow of standard addition; however, this would increase the number of samples to be analyzed. Another drawback to this approach would be the increased requirement of standard substances, which are a limited and cost-prohibitive resource. When conducting CTX investigations in the absence of specific standard compounds, congeners can still be quantified by using the commercially available standards that eluate in the same region of the chromatogram. However, this requires caution, as demonstrated by the example of 52-*epi*-54-deoxyCTX1B and 54-deoxyCTXB, where even minor differences in the retention time (5.85 respective 6.20 min, Figure 2a) can result in pronounced variances in the matrix effects (Table 2). Accordingly, quantitative CTX results should be carefully evaluated. A correction of results by recovery rates determined for a single species might be misleading if different matrices are investigated.

According to the two-tier approach suggested by [13], LC-MS/MS analyses are considered a qualitative confirmation of positive tier-one results rather than a quantitative analysis. By following the EU legislation, “fishery products containing biotoxins such as ciguatoxins […] must not be placed on the market” [18], any positive indication of CTXs violates the EU rule and also falls in line with the qualitative analysis for CTXs applied by the US FDA. Subsequently, quantitation of CTXs is not the major sticking point for CTX diagnostics. Rather the risk of a false negative or false positive result lies with an unequivocal identification of the compound. Efforts should be directed towards synthesizing CTX standards (whenever possible also isotope-labeled compounds) to provide a broad range of congeners for compound confirmation and, in the future, also quantitation by means of certified standards [33].

CTXs are stable during heating and are not decomposed during freeze storage or under mild acidic or basic conditions [15,45,46]. Thus, a transformation or degradation of CTXs in the extracts is not expected. In contrast to that, matrix components might alter during storage, and these alterations could influence the analysis if they were accompanied by changing matrix effects. The results for matrix effects, recovery rates, and extraction efficiencies within 26 days of storage corresponded to 70 to 130% of the values of day 0 without any clear temporal trend (Table 2). The storage of final extracts at −20 °C did not impair CTX analysis, and the extracts did not need to be analyzed on the day of preparation.

#### 2.2.2. Limit of Detection, Limit of Quantitation, and Linearity

**Figure A3:**
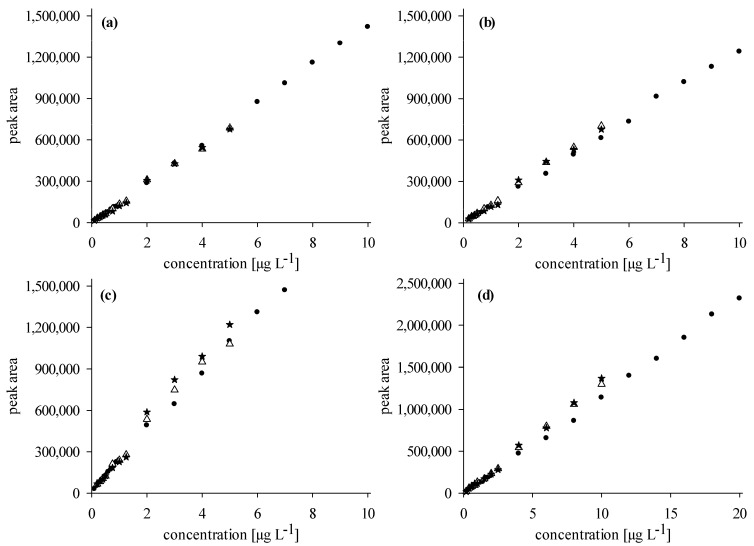
Calibration curves of (**a**) CTX1B, (**b**) 52-*epi*-54-deoxyCTX1B, (**c**) 54-deoxyCTX1B, and (**d**) CTX3C in standards prepared in methanol (black circle), blank matrix methanol extracts of snapper fillet (white triangle), and freeze-dried snapper (red star); details concerning the matrix standard preparation are provided in Section 4.4.2.

Linearity was determined for both methanol standard solutions and matrix samples. Linear calibration functions showed correlation coefficients R^2^ > 0.99 in all cases. For 54-deoxyCTX1B, linearity was not proven for the entire concentration range in methanol (Table 3). According to the slope of the calibration curve, this compound showed the highest response and the most intense signals in the LC-MS/MS chromatograms (Figure 2, calibration curves shown in Figure A3), suggesting a higher ionization efficiency. This probably led to an increasing detector saturation for the most concentrated standard solutions.

LOD and LOQ in the matrix were determined in diluted extracts obtained from fortified fish samples (see Section 4.4.2). Thus the determined limits included and accounted for losses during extraction and sample clean-up, as well as matrix effects during LC-MS/MS analyses. The impact of the sample clean-up is most pronounced for CTX3C, as this analyte was observed to split into two fractions (eluate/filtrate 2:3; Figure A2). Despite this, the determined LOD and LOQ values were still comparable to congeners appearing in the eluate fraction only (Table 3), making the method suitable also for CTX3C analysis.

In the literature, LOD/LOQ are often determined for fortified blank extracts. In that case, values include matrix effects but no analyte losses during sample preparation. Taking into account values for matrix effects and recovery rates (Table 2), LOD/LOQ presented in Table 3 should be corrected by approximately factor 0.6 to obtain values only impaired by matrix effects (e.g., the results for CTX1B in snapper fillet on day 0 correspond to a correction factor of 0.63). However, this assumes that recovery rates are constant for the entire concentration range. The comparability of LOD/LOQ values is also affected by the approach utilized for their determination. For instance, values can be estimated by the blank sample method (calculation of the noise’s standard deviation in a blank sample at the analyte’s retention time), by the calibration approach using standards in the lower concentration range, and by S/N as performed here [47]. Depending on the method, differences up to factor 2 were reported [48].

The number of transitions recorded has an influence on the LOD/LOQ as well. Values were improved by factor 2 when the number of transitions was reduced from twenty down to three (Table 3). Using a reduced number of transitions, 0.01 µg kg^−1^ CTX1B can be detected in snapper fillet samples. Limits could be further improved by acquisition tools such as retention time-dependent MRM (if retention times are known) or by increasing the dwell time (scan time). To enable the comparability between published methods, these parameters should be provided along with information regarding how the values were calculated and whether limits were determined for extracts fortified before analysis or for fish samples fortified before sample preparation.

Taking these complications into account, values obtained for the enzyme method are considered comparable to data published recently [23,25,48]. The optimization of electrospray source parameters might further enhance the limits, but currently, this step is hampered by the limited standard availability.

#### 2.2.3. Blank Matrix Samples

Blank matrix extracts were prepared (Section 4.2 and Section 4.4.1) and were analyzed both using sodium and ammonium adducts as precursor ions (Section 4.3) for all three fish species and both tissue type matrices in order to evaluate the selectivity of the methods. The filtrate samples revealed several peaks, mainly between 5 and 8 min (Figure A1). Intensities were higher in the fillet than in the freeze-dried samples. In the matrix derived from parrotfish (*S. ghobban*), the lowest number of peaks were observed (Figure A1).

**Figure A4:**
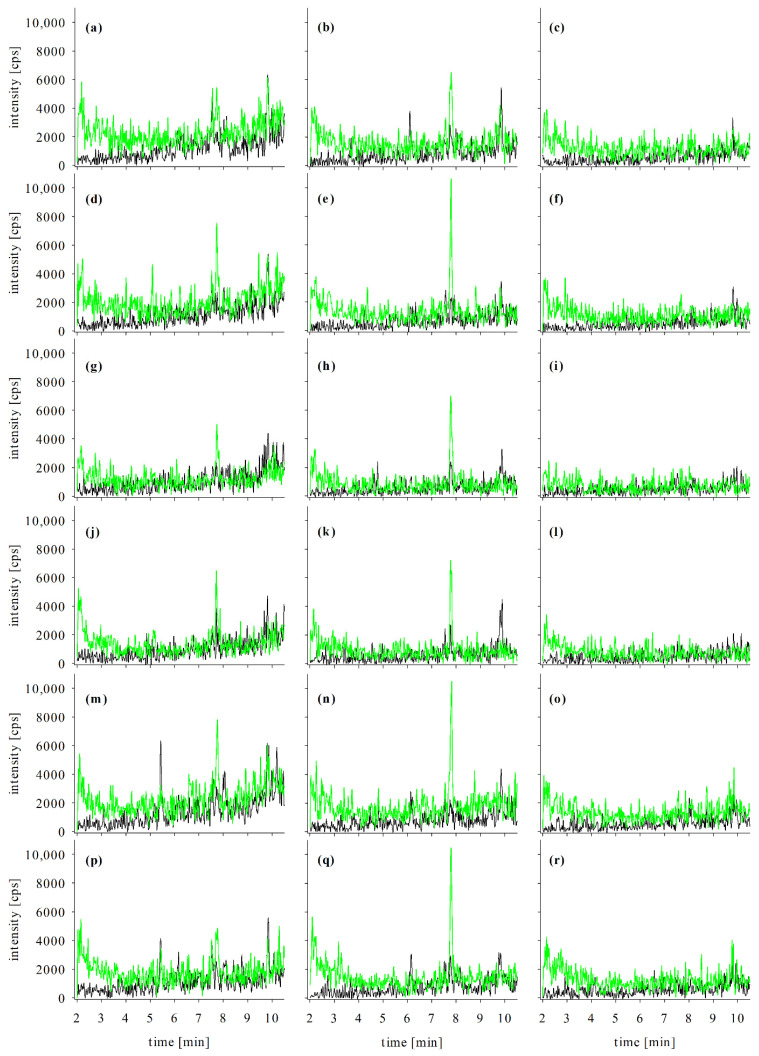
Total Ion Count chromatograms (TIC) of blank matrix eluate (green) and filtrate (black) extracts of (**a**–**c**) snapper fillet, (**d**–**f**) snapper freeze-dried, (**g**–**i**) parrotfish fillet, (**j**–**l**) parrotfish freeze-dried, (**m**–**o**) grouper fillet, and (**p**–**r**) grouper freeze-dried with left, middle, and right columns showing TIC of CTX3C-group, CTX4A-group, and C-/I-CTX congeners, respectively; analyses were performed for product ions of ammonium adducts according to Section 4.3.2; peaks at 7.85 min in the eluates are caused by the product ion [M + H]^+^ of CTX3C (left column) and CTX4A (middle column) only.

For the fortified samples, CTX3C was found in both the filtrate and the eluate fraction, whereas the other congeners investigated were exclusively detected in the eluate. CTX congeners that are more polar than CTX3C are expected to pass into the eluate. Less polar congeners (e.g., CTX4A) are expected to pass (mainly) into the filtrate fraction. Therefore, most peaks observed in the filtrate fraction of the blank samples are ascribed to matrix components possessing a similar *m*/*z* as the respective congeners. This assumption is supported by the analyses of the ammonium adducts, which revealed no peaks for filtrate samples (Figure A4).

LC-MS/MS chromatograms of sodium adducts showed an intense signal at 7.85 min for both eluate and filtrate extracts. Blank methanol injections did not show a respective peak. The signal was mainly derived from the *m*/*z* of M-*seco*-CTX4A/B (Figure A1g,h), but the respective product ions of this congener were not detected for the ammonium adducts (Figure A4). The peak was probably derived from solvent constituents or contaminants of the SPE column material that are concentrated during the solvent evaporation steps.

Besides fragmentation, peak identification can be performed based on retention time. For most CTX congeners, no standards are available to check this parameter. However, studies of several research groups provide information about the elution profile of the respective congener groups (Table 4 and Table 5). The data indicate where the congeners should elute in a described method or setup. They could be used as a decision-type workflow for consideration and could help to minimize potential false-positive conclusions.

For example, based on these available data, M-*seco*-CTX4A/B should elute close to the peak of 52-*epi*-54-deoxyCTX1B (5.9 min, Table 4) and, therefore, an elution at 7.85 min is considered unlikely. The same decision tree would exclude signals at *m*/*z* of 52-*epi*-54-deoxyCTX1B, 54-deoxyCTX1B, CTX3C (retention times also known for these compounds, Table 4), I-CTX-5, and 51-hydroxyCTX3C, to represent CTX congeners at 7.85 min (Figure A1g,h).

Chromatograms of the confirmation analyses revealed only two peaks in the eluates deriving from the MRM transition of the [M + H]^+^ of CTX3C and CTX4A (Figure A4). The presence of the compounds can be excluded based on the retention time (7.85 min) and the lack of other product ions (detailed discussion on the aspect of peak identification in Section 2.3).

Consequently, the results for the six matrices investigated herein highlight the broad applicability of the enzyme method for CTX analyses. Matrix constituents do not interfere with the qualitative detection of CTX, demonstrating the selectivity of the method.

#### 2.2.4. Repeatability

A freeze-dried snapper sample (*L. bohar*), naturally contaminated with CTXs, was extracted according to the enzyme protocol (fourfold preparation; Section 4.2), and the extracts were analyzed using sodium adducts (duplicate injection; Section 4.3.1). The repeatability (expressed as relative standard deviation) was determined for several congeners present in the sample. Details concerning peak identification and assignment are discussed in Section 2.3.

In total, eight peaks were utilized for the evaluation, namely 2,3,51-trihydroxyCTX3C, 2,3-dihydroxyCTX3C (two peaks), 51-hydroxyCTX3C, M-*seco*-CTX3C (two peaks), and 2-hydroxyCTX3C (two peaks). Repeatability ranged from 4.9% for 2,3-dihydroxyCTX3C (peak at 5.25 min) to 11.3% for 51-hydroxyCTX3C. Nagae et al. (2021) reported repeatability between 2.3% and 5.3% for two congeners (CTX1B and CTX3C) and matrices (snapper and grouper) fortified at a level of 0.1 ppb [23]. The contents of the congeners investigated in this study cannot be estimated so far; thus, comparability is limited at this point. It also has to be taken into account that, for this part of the validation, naturally contaminated material was used, which might lead to higher repeatability values compared to fortified material. For a naturally contaminated matrix, a release of internally bound analytes is required. In the case of fortified samples, analytes are externally applied and can be extracted from the matrix surface. This might facilitate the extraction in the latter case.

RSD values obtained are below 20%, reflecting good repeatability of the method, according to SANTE/12682/2019 [43]. Therefore, the enzyme protocol has been demonstrated as suitable for performing single determinations. According to a study by Oshiro et al. (2021), CTXs are almost equally distributed within the fillet, so the error of sampling or inhomogeneity should not impair the results in the case of a single determination [51].

### 2.3. Confirmation Analyses in Naturally Contaminated Samples

CTX standards, synthesized or isolated from contaminated material, are rare, and unequivocal peak identification remains challenging in CTX analyses, resulting in a semi-targeted approach for most congeners (known molecular mass, no reference standard for proving retention time and fragmentation pattern). Nevertheless, the monitoring of (specific) product ions is a valuable tool for substance identification. According to SANTE/12682/2019 or the Commission Implementing Regulation (EU) 2021/808, at least two product ions should be recorded [43,52]. Besides the presence of the two product ions, their ratio has to be taken into account. The ion ratio is ideally compared to a standard compound, and the ratio in the sample may only deviate within a defined range (e.g., <30% based on SANTE/12682/2019).

In the case of CTXs, sodium adducts are stable and undergo almost no fragmentation or sensitivity loss even when exposed to high collision energies (this study, [49]). In contrast to that, ammonium adducts can be easily fragmented. Intense product ions derive from the cleavage of ammonium, resulting in the [M + H]^+^ ion, followed by the loss of one or more water molecules as typically described for CTXs (early reports by, e.g., [10,17,46]).

#### 2.3.1. Generation and Fragmentation of Ammonium Adducts

**Figure A5:**
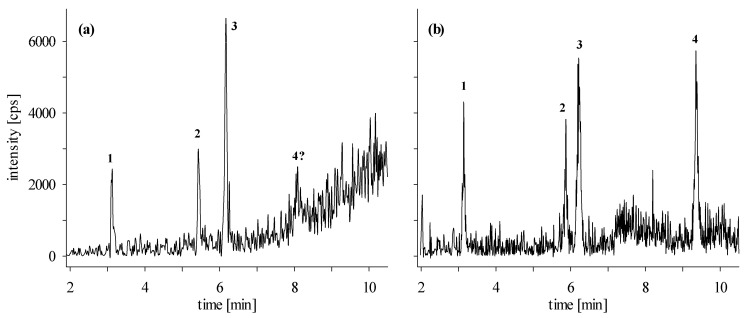
Total Ion Count chromatograms (TIC) of CTX standard in methanol with 1.3 µg L^−1^ CTX1B (1), 52*-epi-*54-deoxyCTX1B (2), 54-deoxyCTX1B (3), and 2.7 µg L^−1^ CTX3C (4) with (**a**) acetonitrile (dwell time 50 ms) and (**b**) methanol/acetonitrile 3:1 (*v*/*v*) as eluent B (dwell time 30 ms); TICs correspond to the sum of ammonium adducts’ product ions ([M + H]^+^, [M + H − H_2_O]^+^, [M + H − 2H_2_O]^+^, [M + H − 3H_2_O]^+^); *m*/*z* are provided in Table 1; gradient for (**a**) started with 50% B; proportion of B was increased to 95% within 9 min, and this ratio was kept for 1 min; within 1 min, the ratio was set back to initial conditions, the column was allowed to equilibrate for 3 min (total run time 14 min); for both analyses, the following parameters were utilized: ion spray voltage 5500 V, source temperature 250 °C, gas 1 60 psi, gas 2 50 psi, curtain gas 40 psi, DP 70 V, EP 6 V, CXP 12 V, CE 30 eV, and source position (horizontal/vertical) 5.0/5.0 (parameters differ from Section 4.3.2 as the analyses were performed before the final parameter optimization).

The formation of ammonium adducts is described to be solvent-dependent. Methanol is supposed to promote the formation of sodium adducts, whereas acetonitrile supports the formation of pseudo molecular ions [M + H]^+^ or ammonium adducts [25,49]. With the setup utilized in this study, acetonitrile was found to suppress the ammonium adduct formation of CTX3C (Figure A5), so no pure acetonitrile eluent was utilized. The temperature was found to be a relevant parameter for adduct formation, as it was described for other analytes [53]. For the QTrap system utilized in this study (Sciex QTrap 6500+), electrospray source temperatures above 400 °C led to an almost exclusive formation of sodium adducts. Below 300 °C, ammonium adduct formation was observed for all four congeners, but sodium adducts were still detectable as well. Additionally, the ion spray source’s geometry had an impact on adduct formation. Almost no sodium adduct formation was observed at 400 °C using the Time-of-Flight mass spectrometer (ToF-MS), but signals of the ammonium adducts were detected. These examples underline the complexity of CTX analyses, as the usually exhaustive tuning of source parameters cannot be conducted due to missing or limitedly available standard compounds.

As the detection of ammonium adducts and their respective product ions is applied for confirmation only, no full method validation was conducted. Based on the LOD samples (Section 4.4.2), ammonium adduct detection was estimated to be factor 4 to 10 less sensitive than the sodium adduct method (see also Figure 2). ToF-MS are known to be less sensitive than triple quadrupole systems [48,54]. Consequently, methods utilizing a ToF-MS cannot achieve LODs comparable to triple quadrupole systems. As both ammonium adducts and sodium adducts were formed under the conditions employed for low-resolution ammonium adduct analyses, the LODs of the confirmation method was accordingly higher.

CTX method development often focusses on the detection of sodium adducts, as the best LOD can usually be achieved that way. Methods solely including sodium adducts can risk delivering false positive results, particularly when a matching standard is not available for confirmation. Therefore, CTX confirmation studies should consider the detection of product ions or high-resolution analysis as well. Strictly speaking, the use of LC-MS/MS for confirmation of CTXs in a sample is limited not only by the LOD of a method monitoring sodium adducts but also by the sensitivity of the confirmatory method (limitation of a false-positive results).

Using fragmentation of ammonium adducts for confirmation provides valuable information for peak identification even if standards are not available (semi-targeted approach). In this study, four compounds were utilized. These differ in their oxidation statuses, with CTX3C being a low oxidized algal metabolite [4]. CTX1B, 52-*epi*-54-deoxyCTX1B, and 54-deoxyCTX1B are transformation (oxidation) products of CTX4A with ratios depending on the fish’s position in the food web and the species’ enzyme pool [24,55]. These differences in the oxidation status are reflected in the fragmentation pattern. For CTX3C, [M + H]^+^ and [M + H − H_2_O]^+^ were the dominant product ions, whereas the product ion deriving from threefold water loss was only detected in trace amounts (Figure 2b).

In the case of 52-*epi*-54-deoxyCTX1B and 54-deoxyCTX1B, the dominant product ion was [M + H − H_2_O]^+^, followed by [M + H − 2H_2_O]^+^; [M + H]^+^ and [M + H − 3H_2_O]^+^ showed similar, lower intensities. Belonging to the CTX4A-group, these congeners possess a hydroxylated side chain (in contrast to CTX3C), which favors the multiple cleavages of water. CTX1B contains an additional hydroxyl group, and for this congener, product ions deriving from losses of one, two, and three water molecules revealed the same intensities (Figure 2b). Based on these observations, higher oxidized congeners should deliver product ions of multiple water loss. Furthermore, congener groups with and without an (oxidized) side chain can be distinguished. If potential peaks of unknown congeners shall be identified, the relative intensity of the respective product ions should be taken into account as a type of ‘plausibility check’. As congeners show different fragmentation patterns, several product ions should be included in the detection methods as focusing only on, e.g., [M + H − H_2_O]^+^, and [M + H − 2H_2_O]^+^ might omit several congeners. Confirmation methods employed in this study monitored four product ions for all compounds ([M + H]^+^, [M + H − H_2_O]^+^, [M + H − 2H_2_O]^+^, and [M + H − 3H_2_O]^+^).

Retention time is another parameter utilized for compound confirmation and identification. As already discussed for blank matrix samples (Section 2.2.3), data available from a literature search can give an indication of the order of congeners eluting in the individual chromatographic setup (Table 4 and Table 5).

#### 2.3.2. Confirmation Analyses in Naturally Contaminated Samples

**Figure 3:**
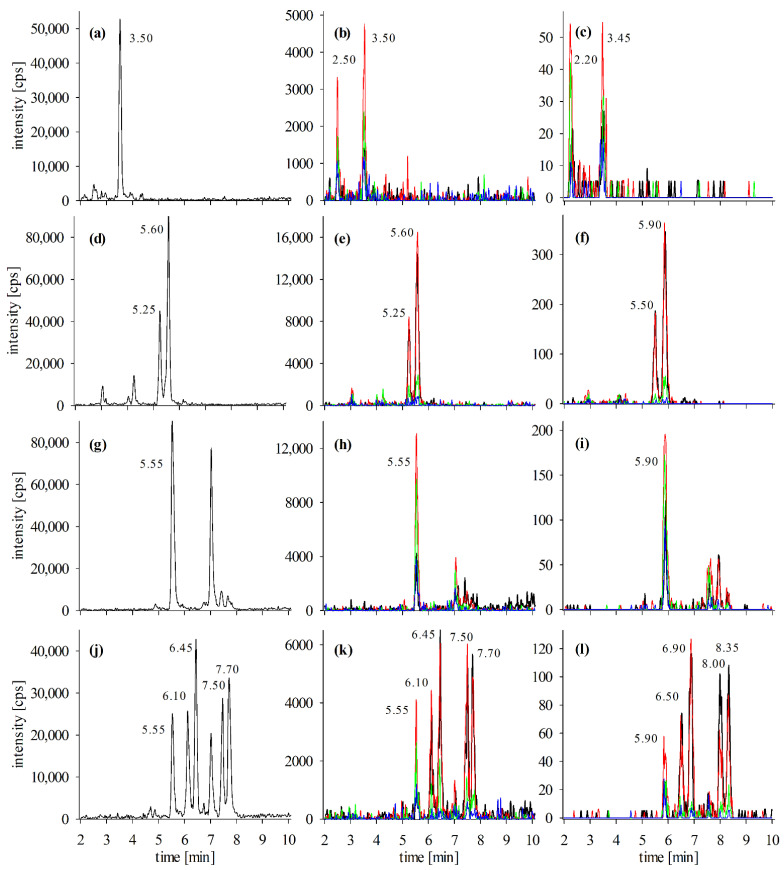
Extracted Ion Chromatograms of the LC-MS/MS analysis of a contaminated snapper fillet (*L. bohar*) containing potential CTX-congeners with (**a**–**c**) 2,3,51-trihydroxyCTX3C, (**d**–**f**) 2,3-dihydroxyCTX3C, (**g**–**i**) 51-hydroxyCTX3C, and (**j**–**l**) M-*seco*-CTX3C respective 2-hydroxyCTX3C; the left column shows the analysis of the sodium adducts [M + Na]^+^, the middle and right columns show the low-resolution and high-resolution analyses of the ammonium adducts, respectively, with the product ions [M + H]^+^ (black), [M + H − H_2_O]^+^ (red), [M + H − 2H_2_O]^+^ (green), and [M + H − 3H_2_O]^+^ (blue); *m*/*z* are provided in Table 1; retention times are provided for the potential CTX congeners according to the discussion in the main text.

During a CP incident in Germany in 2017, contaminated snapper fillet samples were obtained. One fillet with CTX-like activity in the N2a-assay was extracted according to the enzyme protocol and analyzed via LC-MS/MS. First, extracts were analyzed via sodium adduct monitoring (Section 4.3.1), and several peaks of potential CTX congeners were detected. To distinguish between matrix and congener peaks, the putative congeners were analyzed using ammonium adduct fragmentation (low- and high-resolution, Section 4.3.2 and Section 4.3.3). The evaluation was performed based on the obtained LC-MS/MS data, combined with retention time data from the literature (Figure 3, Table 4 and Table 5). 

Using this approach, several potential congeners of the CTX3C-group were identified, namely 2,3,51-trihydroxyCTX3C, 2,3-dihydroxyCTX3C, 51-hydroxyCTX3C, M-*seco*-CTX3C, and 2-hydroxyCTX3C (Figure 3). Peaks of these compounds were detected with all three analytical methods, proving the presence of CTX congeners. However, for the final compound, identification reference standards are needed.

Based on literature data, 2,3,51-trihydroxyCTX3C is expected to possess a retention time similar to CTX1B (3.1 min and 3.0 min in the low-resolution and high-resolution setup, respectively; Table 4). Therefore, the peak detected at 3.50 min, respective 3.45 min, was assigned to this compound (Figure 3a–c). The congeners 2,3-dihydroxyCTX3C and 51-hydroxyCTX3C should possess similar retention times, and their elution should occur close to 52-*epi*-54-deoxyCTX1B (Table 4). These aspects are fulfilled by the assigned peaks in the corresponding LC-MS/MS chromatograms (Figure 3d–i).

M-*seco*-CTX3C and 2-hydroxyCTX3C possess the same molecular formula and cannot be distinguished by high-resolution MS (Table 1). According to the retention times reported in the literature (Table 4), peaks at 6.10/6.45 min are ascribed to M-*seco*-CTX3C and peaks at 7.50/7.70 min to 2-hydroxyCTX3C (Figure 3j–l). According to literature data, M-*seco*-CTX3C should elute before 52-*epi*-54-deoxyCTX1B (Table 4). This was not the case in this study, as M-*seco*-CTX3C showed a higher retention time than the other congener. Differences are ascribed to various column materials utilized which can slightly change the elution profile (see, e.g., data for 2,3-dihydroxyCTX3C in Table 4). However, both studies found M-*seco*-CTX3C to elute before 2-hydroxyCTX3C; thus, the peak assignment was performed as mentioned above. The peak at 7.70 min was not detected in blank matrix samples (Figure A1g,h) and is consequently not part of the matrix peak detected in all extracts at 7.85 min, but derived from a potential CTX congener. The peak at 7.00 min, detected for sodium adducts only, probably derives from matrix compounds as no intense product ions were detected for ammonium adducts at this retention time. This highlights the importance of confirmation analyses besides the detection of sodium adducts.

**Figure A6:**
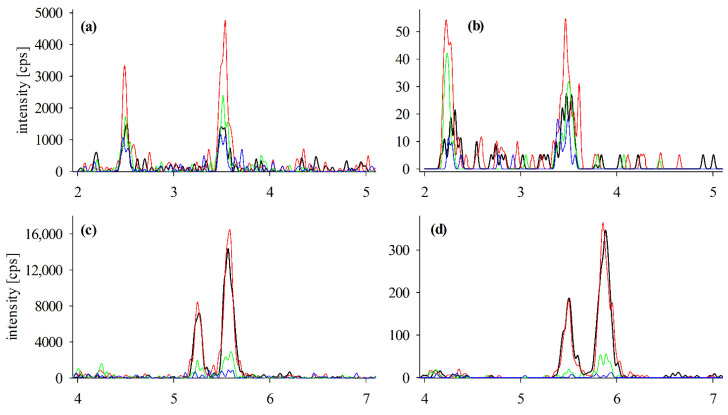

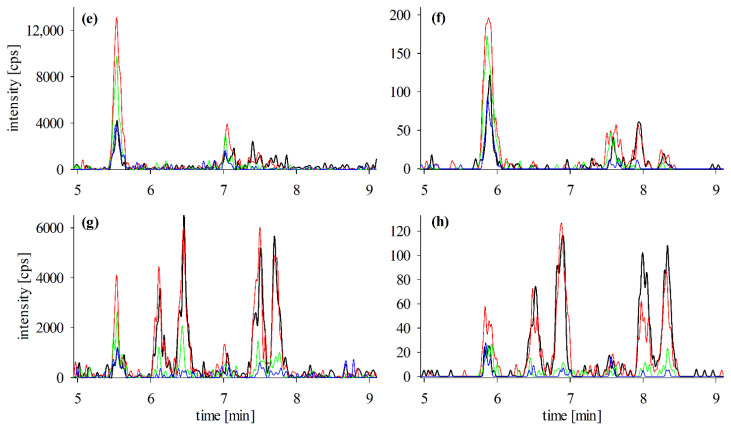
Enlarged Extracted Ion Chromatograms of the LC-MS/MS analysis of a contaminated snapper fillet (*L. bohar*) containing potential CTX-congeners with (**a**,**b**) 2,3,51-trihydroxyCTX3C, (**c**,**d**) 2,3-dihydroxyCTX3C, (**e**,**f**) 51-hydroxyCTX3C, and (**g**,**h**) M-*seco*-CTX3C respective 2-hydroxyCTX3C; left and right columns show the low-resolution and high-resolution analysis of the ammonium adducts, respectively, with the product ions [M + H]^+^ (black), [M + H − H_2_O]^+^ (red), [M + H − 2H_2_O]^+^ (green), and [M + H − 3H_2_O]^+^ (blue); *m*/*z* are provided in Table 1.

The peak at 5.55 min was not identified as 2-hydroxyCTX3C or M-*seco*-CTX3C, although the peak was present in all chromatograms and fragmentation was observed (Figure 3j–l). As discussed above, the ion ratio of the detected product ions has to be considered for peak identification. For the potential peaks of M-*seco*-CTX3C and 2-hydroxyCTX3C at 6.10/6.45 and 7.50/7.70 min, [M + H]^+^ and [M + H − H_2_O]^+^ were the most intense product ions (Figure 3k,l), whereas the peak at 5.55 min showed [M + H − H_2_O]^+^ and [M + H − 2H_2_O]^+^ as dominant product ions, independent of the analytical setup. The latter fragmentation pattern was also observed for 51-hydroxyCTX3C (Figure 3h,i; enlarged graphs in Figure A6). This example revealed another pitfall in the analysis of CTXs. Many congeners differ by 2 amu only, e.g., 2- and 51-hydroxyCTX3C (Table 1). Due to the high number of carbon and oxygen atoms, isotopic peaks become relevant in CTX MS analysis. For 51-hydroxyCTX3C, the isotope peaks of M, M + 1, M + 2, have an intensity of approximately 100:65:24. The [M + H − H_2_O]^+^ product ions of 51- and 2-hydroxyCTX3C at 5.55 min possess a peak ratio of 100:30.8, which falls into the relative ion ratio tolerance of 30% according to SANTE/12682/2019 [43] (100:24 corresponds to the range of 100:16.8 to 100:31.2). Thus, the peak at 5.55 min in the 2-hydroxy/M-*seco*-CTX3C chromatograms is ascribed to the M + 2 isotope peak of 51-hydroxyCTX3C. The isotope problem can be overcome by using high-resolution MS with a narrow extraction window as *m*/*z* of 2-hydroxyCTX3C, and isotope peaks of 51-hydroxyCTX3C differ by approximately 15 ppm. Due to the low peak intensity of some product ions, an extraction window of ±25 mDa, respective 24 ppm, was utilized in this study. For more intense signals, the window could be reduced to exclude isotope detection. For low-resolution MS, the ion ratio should be carefully considered to prevent a false-positive peak assignment. 

**Figure A7:**
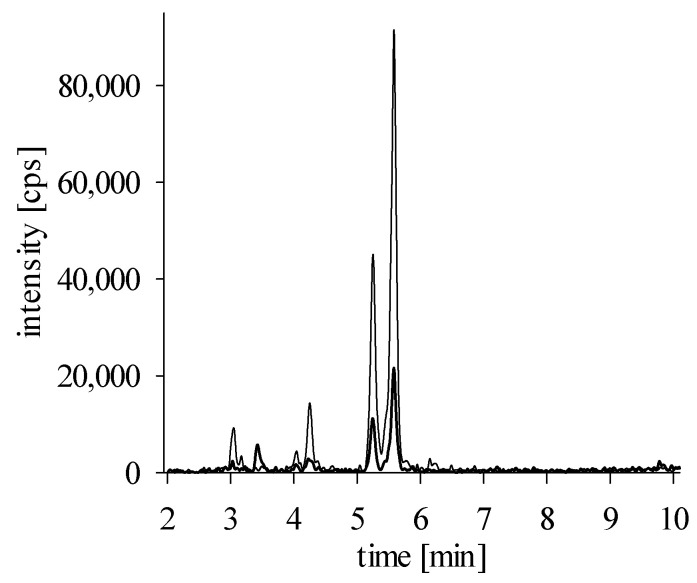
Extracted Ion Chromatograms of the LC-MS/MS analysis of a contaminated snapper (*L. bohar*) fillet containing potential CTX-congeners with 2,3-dihydroxyCTX3C (thin line) and A-*seco*-51-hydroxyCTX3C (bold line); values for *m*/*z* are provided in Table 1.

Peaks of potential A-*seco*-51-hydroxyCTX3C revealed exactly the same retention times as peaks of 2,3-dihydroxyCTX3C (Figure A7). A-*seco*-51-hydroxyCTX3C is supposed to elute before 2,3-dihydroxyCTX3C (Table 4), so no further investigations were performed in the case of that congener. Observed peaks at 5.25 and 5.60 min are regarded as isotope peaks of 2,3-dihydroxyCTX3C as both compounds differ by 2 amu. One low-intensity peak was observed at 3.45 min, which would correspond to A-*seco*-51-hydroxyCTX3C based on the expected retention time, but detection of two product ions within the confirmation methods was not possible.

With the exception of 51-hydroxyCTX3C, all congeners showed two defined peaks in the chromatograms of the ammonium adduct’s product ions (Figure 3). These might derive from epimeric forms (e.g., 49-*epi*-2,3-dihydroxyCTX3C and 2,3-dihydroxyCTX3C at 5.25 and 5.60 min, respectively; Figure 3d,e). One possible reason could be the addition of acetic acid to SPE solvents, as acidic conditions favor epimerization [14]. However, the same fillet samples were extracted using a protocol without acidic solvents (method based on [22]), and multiple peaks were detected in those samples as well. Epimers are probably already present in the fish matrix rather than the result of a formation during sample preparation, as already discussed (see Section 2.2.1).

### 2.4. Extract Suitability for In Vitro Assay (N2a-Assay) Analysis

The development of the enzyme protocol focused on the optimization for LC-MS/MS analysis. However, in vitro type assays are commonly employed during CP incidents for CTX analysis (overview provided, e.g., by [7]), but also within CP prevention programs (e.g., on the Canary Islands, [56]); therefore, the extracts were also investigated for suitability when being applied to the N2a-assay.

**Figure 4:**
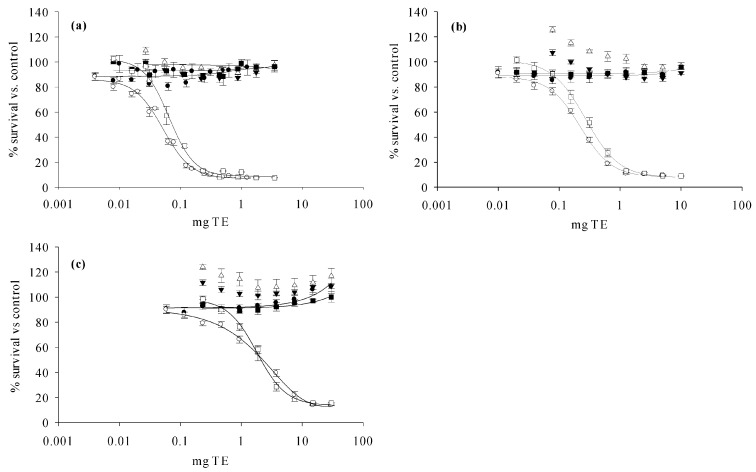
Dose-response curves of extracts obtained for naturally contaminated *L. bohar* samples of (**a**) high, (**b**) medium, and (**c**) low toxicity with filtrate (triangles), eluate (squares), and combination of filtrate and eluate (circles) tested; open and black symbols show samples with and without ouabain/veratridine (OV+/OV−) addition, respectively; data points and error bars are based on a minimum of three independent 96-well plate analyses and triplicate wells per data point per plate/sample.

No adverse effect on cell viability (growth or death) was observed when compared to an unexposed control (with or without the addition of ouabain/veratridine) when applying a blank matrix extract of either snapper fillet or freeze-dried snapper at a dosage of up to 15.62 mg wet TE and 1.56 mg dry TE; independent of which portion of the final extract was used (eluate, filtrate, or combination). Beyond these concentrations, an increase in cell viability (growth of approximately +15% above the control) was observed beginning at 31.25 mg wet TE and 3.13 mg dry TE. Other studies investigating matrix interference effects on the N2a-assay recommended a maximum matrix load of 4.6 mg TE (20 mg TE mL^−1^) to avoid potential matrix interferences [57]. However, the optimum maximum tissue dose equivalent (MTDE) was found to be species-dependent, and the lipid content was considered as a relevant factor. For fish with a low- and medium-lipid content, an MTDE of 50 mg TE was proposed, whereas, for high-lipid content fish, a limit of 5 mg TE was applied [41]. In accordance with those results, no matrix effect was evident in the naturally incurred sample extracts when 30 mg wet TE was used (Figure 4c). Since the type of fish can impact the cell assay response in species-specific (i.e., matrix) ways, the blank reference fish (*L. malabaricus*) may be slightly less suitable for the assay than the naturally incurred species (*L. bohar*) utilized here.

**Figure A8:**
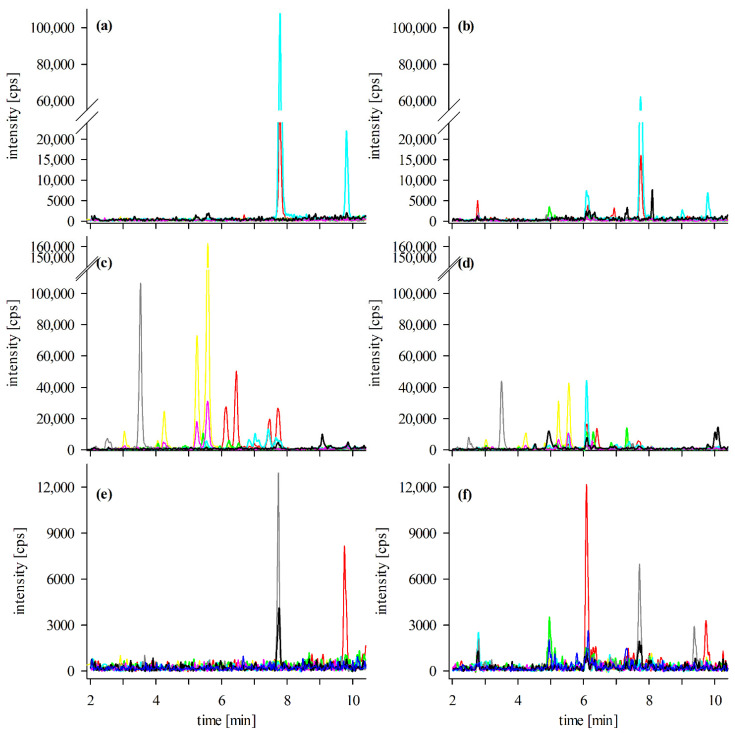
Extracted Ion Chromatograms (EIC) of the LC-MS/MS analysis of a contaminated snapper fillet (*L. bohar*) containing potential congeners of the CTX3C group (see Section 2.3.2 for details) extracted using both the enzyme protocol (Section 4.2, left column) and the method described by [22] (Section 4.5, right column); intensities in this figure and Figure 3 differ as extracts of two different fillet samples were utilized; EIC correspond to twenty transitions of the sodium adducts [M + Na]^+^ recorded within one analytical run (Section 4.3.1) showing congeners of the (**a**,**b**) CTX4A group, (**c**,**d**) CTX3C group, (**e**,**f**) C-CTX, and I-CTX group; color codes are (**a**,**b**) CTX4A group: black—CTX4A/B, blue—M-*seco*-CTX4A/B, red—52-*epi*-54-deoxyCTX1B and 54-deoxyCTX1B, green—CTX1B and epimers/isomers (see Table 1), yellow—7-oxoCTX1B, pink—7-hydroxyCTX1B, grey—4-hydroxy-7-oxoCTX1B, (**c**,**d**) CTX3C group: black—CTX3C/B, blue—51-hydroxyCTX3C, red—2-hydroxyCTX3C and M-*seco*-CTX3C, green—51-hydroxy-2-oxoCTX3C, yellow—2,3-dihydroxyCTX3C, pink—A-*seco*-51-hydroxyCTX3C, grey—2,3,51-trihydroxyCTX3C, (**e**,**f**) C-CTX and I-CTX group: black—C-CTX-1/2 and I-CTX-1/2, blue—C-CTX-3/4, red—C-CTX reaction product 8, green—C-CTX reaction product 9, yellow—C-CTX-1127, pink—C-CTX-1157 and I-CTX-3/4, grey—I-CTX-5, dark blue—I-CTX-6; *m*/*z* are provided in Table 1; the discussion about matrix peaks is provided in Section 2.2.3; please note that the sample concentrations differ by factor 2 as the final samples were reconstituted in 500 µL (enzyme protocol, left column) and 1000 µL methanol (right column).

Extracts of naturally contaminated material delivered dose-response curves suitable for the calculation of an EC_50_ (Figure 4). Eluate and the combination of eluate and filtrate showed comparable results with an EC_50_ of 0.066 and 0.052 mg wet TE (high toxicity), 0.285 and 0.278 mg wet TE (medium), and 1.83 and 2.26 mg wet TE (low), respectively. These data were similar to the EC_50_ values determined for extracts prepared with an established extraction method (described by [22]) from the same sample material (0.055, 0.268, and 2.17 mg wet TE, respectively). This implies comparable extraction efficiencies of the established method and the new enzyme protocol presented here, making the enzyme treatment prior to extraction a suitable alternative to mechanical treatment. Furthermore, enzymatic digestion does not lead to an increased number of matrix peaks enabling the identification of CTX congeners even in the absence of reference standards (Section 2.3., comparison of LC-MS/MS chromatograms obtained for both extraction methods provided in Figure A8). Therefore, the enzyme protocol results in extracts that are suitable for analysis by both a functional bioassay (N2a-assay) and an instrumental analytical method (LC-MS/MS), which, in combination, are commonly applied for CP response analysis and diagnostic support. One extraction that can be utilized for two individual methods of analysis maintains a simplified workflow for the commonly used two-tier approach for CTX analysis when providing analytical confirmation of CP events.

## 3. Conclusions

A novel sample preparation protocol for CTX analysis in fish was developed and (partly) validated, including an enzymatic break-down of the fish tissue, followed by extraction, defatting, RP, and NP SPE. Based on recovery rates and matrix effects, the method was proven applicable to different fish species, as well as fillet and freeze-dried matrices. For CTX1B, contents of 0.01 µg per kg wet weight could be detected, making the procedure also suitable for low concentration samples. Furthermore, extracts were found suitable for application in the N2a-assay so the enzyme protocol can be used for the suggested two-tier workflow for CTX suspected samples. LC-MS/MS analyses were conducted as screening analyses of the sodium adducts for >30 CTX congeners described in the literature, followed by low- and high-resolution mass spectrometry confirmative analyses using the respective ammonium adducts and their product ions. Including a broad range of congeners, the method can also be utilized for samples with an unknown CTX profile. This approach enabled the identification of CTXs of the CTX3C group in a naturally contaminated sample, even without standard compounds. This new method will be utilized in future studies to investigate samples involved in CP outbreaks, as well as environmental samples from global CP endemic regions. The application of this method to organisms in a biological food web can further elucidate the trophic transfer and metabolic pathways for CTXs in endemic regions, with the ultimate goal of supporting CP risk assessment efforts.

## 4. Materials and Methods

### 4.1. Reagents and Materials

Standard solutions of CTX1B (4 µg L^−1^), 52-*epi*-54-deoxyCTX1B (P-CTX-2, 1 µg L^−1^), and 54-deoxyCTX1B (P-CTX-3, 2 µg L^−1^) in methanol were purchased from Professor R. J. Lewis (The Queensland University, Australia, prepared 17.11.2005). CTX3C (100 ng, lot APK4222) was purchased from FUJIFILM Wako Chemicals Europe GmbH (Neuss, Germany) and dissolved in 1 mL methanol. All solutions were stored in glass vials at −20 °C. Mixed standard solutions were prepared in methanol and stored in glass vials at −20 °C.

Acetonitrile, methanol, formic acid, ammonium acetate (all LC-MS grade), chloroform, ethyl acetate (LC grade), *n*-hexane (GC-MS grade), acetone, acetic acid, citric acid monohydrate, anhydrous sodium carbonate, and sodium chloride (all p.a. grade) were obtained from various suppliers. Papain (>30,000 USP-U mg^−1^, for biochemistry) was purchased from Carl Roth (Karlsruhe, Germany). Deionized water was prepared using a Milli-Q Reference A+ system (Merck Millipore, Darmstadt, Germany). Cartridges for solid-phase extraction (SPE) were obtained from Agilent (Bond Elut SI (silica), 500 mg, 3 mL; Agilent, Waldbronn, Germany) and Macherey-Nagel (Chromabond Easy, 3 mL, 200 mg; Macherey-Nagel, Düren, Germany). According to the manufacturer, Chromabond Easy sorbent consists of a polystyrene-divinylbenzene copolymer modified with a weak anion exchanger.

The fish fillet samples for the method development and validation were purchased at a local wholesale (Berlin, Germany). Matrices included the genera *Lutjanus* or snapper (*Lutjanus malabaricus*), *Scarus* or parrotfish (*Scarus ghobban*), and *Epinephelus* or grouper (*Epinephelus areolatus*). Fillets were utilized without skin. Lyophilization was performed in a freeze-dryer (Lyovac GT2, Amsco/Finn-Aqua, Hürth, Germany) over 36 h for unskinned fillets from the same sample lot. Freeze-dried material was ground to a fine powder, transferred to 50 mL polypropylene tubes, and stored at −20 °C before usage. Water contents were 80% for snapper and grouper and 84% for parrotfish (determined for fillet without skin, glaze water was removed prior to freeze-drying).

Naturally contaminated snapper samples (*L. bohar*, previously verified by DNA barcoding, [58]) were obtained during a CP incident in Germany in 2017. The fish of the respective lot was caught in the FAO mayor fishing area 71. An overview concerning sample data (catchment area, capture time) and previously conducted sample analyses is provided in [58].

Details concerning chemicals, materials, and cell line utilized for the N2a-assay are provided in [59] and Section 4.5.

### 4.2. Sample Preparation—Enzyme Protocol

**Figure 5:**
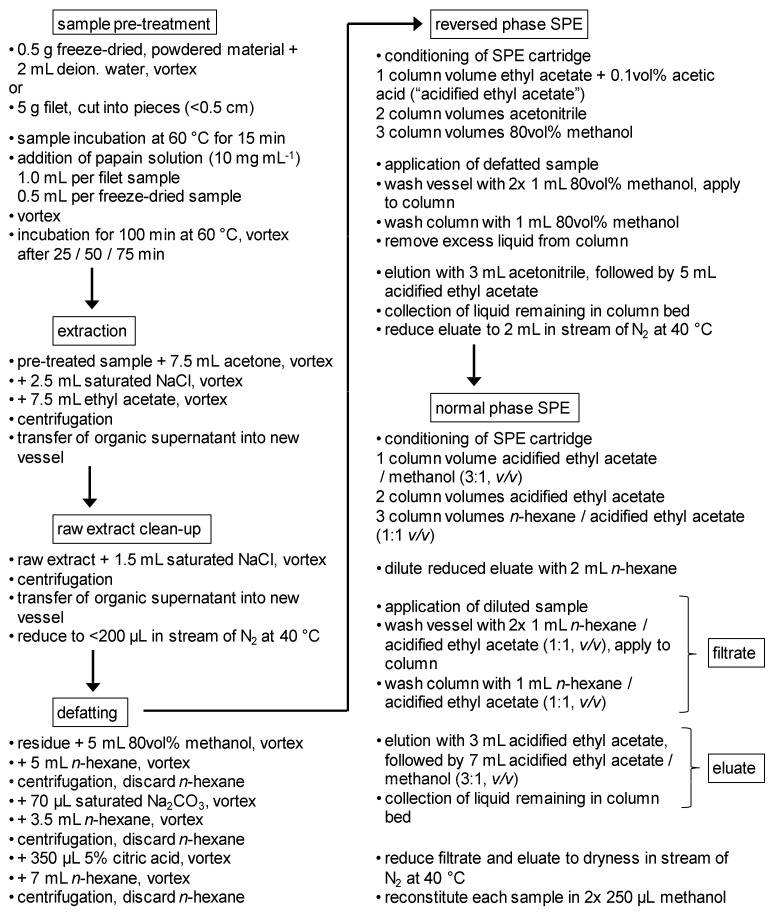
Flow chart of the sample preparation; vortex steps were performed for 30 s each, centrifugation was conducted for 3 min at 1900× *g*, for further details regarding the single steps see Section 4.2.

A flow chart of the sample preparation is provided in Figure 5. Aqueous solutions were prepared with deionized water. Due to limited stability, the papain solution, as well as solvent mixtures for silica gel SPE (SiOH SPE), were prepared right before use. Acidified ethyl acetate (ethyl acetate + 0.1 vol% acetic acid) was prepared freshly on each working day to prevent ester hydrolysis during storage.

Sample preparation was conducted in glass vessels (except SPE cartridges) to avoid the sorption of CTX on plastic surfaces [31]. In the following, vortex steps were performed for 30 s. Centrifugation was conducted at 1900× *g* for 3 min.

#### 4.2.1. Sample Pre-Treatment and Extraction

The fillet (frozen or thawed) was cut into pieces (length ≤ 0.5 cm) and (5.00 ± 0.01) g was weighed and placed into a 50 mL glass vessel with screw cap. For freeze-dried samples, (0.50 ± 0.01) g material was mixed with 2 mL deionized water prior to use.

The samples were incubated at 60 °C for 15 min. Then, papain solution (10 mg mL^−1^) was added at a volume of 1 mL per fillet sample and 0.5 mL per freeze-dried sample, respectively. For protein hydrolysis, samples were incubated at 60 °C for 100 min. To support matrix decomposition, samples were vortexed after 25, 50, and 75 min.

Extraction of CTXs was performed in three consecutive steps. First, 7.5 mL acetone was added to the hydrolyzed sample, and the mixture was vortexed. Then, 2.5 mL of a saturated sodium chloride solution was added, then vortexed. Last, 7.5 mL ethyl acetate was added, followed by vortexing and centrifugation.

The organic supernatant (raw extract) was transferred into a new 50 mL glass vessel. The raw extract was mixed with 1.5 mL saturated sodium chloride solution (vortex), and the solution was centrifuged. The organic extract was transferred into a new 50 mL glass vessel and reduced to ≤200 µL in a stream of nitrogen at 40 °C. In the case where a sample has a high-fat content (e.g., grouper matrix), a liquid fat residue may be present; however, the presence of this residue will not impair the proceeding steps.

#### 4.2.2. Defatting

The defatting procedure was adapted from [23] and modified using reduced solvent volumes (−82.5%) to permit handling in glass centrifuge tubes. Defatting was conducted in three steps. The residue of Section 4.2.1 was reconstituted in 5 mL 80 vol% methanol, and to this, 5 mL *n*-hexane was added. The sample was then vortexed, centrifuged, and the *n*-hexane phase (upper layer) was discarded.

To the methanol phase, 70 µL saturated sodium carbonate solution was added, and the sample was vortexed. Then, 3.5 mL *n*-hexane was added, followed by vortexing, centrifugation, and the *n*-hexane phase was discarded. Last, 350 µL of 5% citric acid solution was added, and the sample vortexed. Then, 7 mL *n*-hexane was added, the sample was vortexed, centrifuged, and the *n*-hexane phase was discarded. The remaining methanol phase was directly utilized for SPE clean-up. Precipitated salt residues were not removed.

#### 4.2.3. Reversed-Phase (RP) SPE

Reversed-phase SPE was conducted using Chromabond Easy cartridges under reduced pressure (ca. 960 mbar). The column was conditioned with one column volume of acidified ethyl acetate, two column volumes of acetonitrile, and three column volumes of 80 vol% methanol.

The defatted sample from Section 4.2.2 was applied to the column with a flow rate of approximately 2 mL min^−1^. The glass vessel was rinsed twice with 1 mL 80 vol% methanol, and each rinse was applied to the column. The column was then washed with 1 mL 80 vol% methanol. Afterward, the column was allowed to run dry to remove excess liquid.

Elution was performed into a single 10 mL glass tube using 3 mL acetonitrile, followed by 5 mL acidified ethyl acetate. Elution was conducted at atmospheric pressure after applying a reduced pressure of 960 mbar to the first approximately 300 µL acetonitrile. In the end, excess liquid remaining in the column bed was collected by applying positive pressure at the column inlet. The eluate was reduced to 2 mL in a stream of nitrogen at 40 °C.

#### 4.2.4. Normal-Phase (NP) SPE

Normal-phase SPE was conducted with Bond Elut SI cartridges at atmospheric pressure. This step provides two fractions (“filtrate” and “eluate”) that contain different CTX congeners and differ in their matrix load. Consequently, the fractions are stored separately.

The column was conditioned with one column volume of acidified ethyl acetate/methanol (3:1, *v*/*v*), two column volumes of acidified ethyl acetate, and three column volumes of acidified ethyl acetate/*n*-hexane (1:1, *v*/*v*). After column conditioning, a 10 mL glass tube was placed under the column outlet for “filtrate” sample collection.

The reduced eluate of Section 4.2.3 was diluted with 2 mL *n*-hexane and applied to the column with a flow rate of approximately 2 mL min^−1^. The glass vessel was rinsed twice with 1 mL acidified ethyl acetate/*n*-hexane (1:1, *v*/*v*), and the rinse solvent was applied to the column. The column was washed with 1 mL acidified ethyl acetate/*n*-hexane (1:1, *v*/*v*). The glass tube under the column outlet was removed afterward. It contained the fraction “filtrate”.

For the elution, an additional separate 10 mL glass tube was placed under the column outlet for collection. Elution was performed with 3 mL of acidified ethyl acetate, followed by 7 mL of acidified ethyl acetate/methanol (3:1, *v*/*v*). In the end, excess liquid remaining in the column bed was collected by applying pressure at the column inlet to obtain the second fraction “eluate”.

Both fractions were reduced to dryness in a stream of nitrogen at 40 °C. For sample reconstitution, the vessels were rinsed twice with 250 µL methanol. Samples were stored in glass vials at −20 °C.

### 4.3. LC-MS/MS Analysis

Three different methods were developed for CTX analysis. Extract screening was conducted via the compounds’ respective sodium adducts. The confirmation of potential CTX congeners was performed via analysis of ammonium adducts and the corresponding product ions, with high- or low-resolution MS. For low-resolution analysis, *m*/*z* of precursors and product ions (Table 1) were adjusted to a unified decimal of x.6 amu in the respective MS methods.

For all methods, 1 mM ammonium acetate +0.5% formic acid (eluent A) and methanol/acetonitrile (3:1, *v*/*v*; eluent B) were used as eluents. Separation was performed at 40 °C using a reversed-phase column Gemini NX-C18 (150 × 2 mm, 3 µm; Phenomenex, Aschaffenburg, Germany).

#### 4.3.1. Analysis of Sodium Adducts [M + Na]^+^

An Agilent 1290 Infinity II UHPLC (Agilent, Waldbronn, Germany) coupled to a Sciex QTrap 6500+ (Sciex, Darmstadt, Germany) was utilized for analysis. Data acquisition and peak integration were performed using the software Analyst 1.6.3.

The separation started with 78% B at a flow rate of 0.45 mL min^−1^. The proportion of B was increased to 92% within 10 min. For column washing, the ratio was increased to 99% within 0.1 min; after an additional 0.5 min, the flow rate was set to 0.60 mL min^−1^. This setting was held for 2.4 min. Then, the flow rate and proportion of B% were reset to initial conditions within 0.2 min, and the system was then allowed to equilibrate for 2.8 min resulting in a total run-time of 16 min. Unless otherwise stated, an injection volume of 2 µL was utilized.

MS analyses were performed in the multiple reaction monitoring modes (MRM) with one transition per congener. Both in Q1 and Q3, the *m*/*z* of the sodium adducts [M + Na]^+^ were selected (Table 1), as these adducts show almost no fragmentation. Due to low-resolution, congeners with similar *m*/*z* were combined in one transition (e.g., 7-oxoCTX1B and C-CTX-1127, M-*seco*-CTX3C methyl acetate, and 51-hydroxy-2-oxoCTX3C). This way, the method included 20 transitions with 50 ms dwell time each.

The following parameters were applied to all congeners: ion spray voltage 5500 V; source temperature 500 °C; gas 1 and 2, each 70 psi; curtain gas 40 psi; declustering potential (DP) 80 V; entrance potential (EP) 6 V; cell exit potential (CXP) 18 V; collision energy (CE) 60 eV; and source position (horizontal/vertical) 5.0/5.0.

#### 4.3.2. Analysis of Ammonium Adducts [M + NH_4_]^+^—Low-Resolution

The same system, gradient, and injection volume (2 µL) as used for [M + Na]^+^-analysis (Section 4.3.1) were applied here.

MS analyses were performed in the MRM mode with four transitions per congener. In Q1, the ammonium adducts [M + NH_4_]^+^, and in Q3, *m*/*z* of the corresponding [M + H]^+^, [M + H − H_2_O]^+^, [M + H − 2H_2_O]^+^, and [M + H − 3H_2_O]^+^ product ions were selected (Table 1). Due to the number of transitions, a maximum of eight congeners was included in one method with a dwell time of 30 ms per transition. For method validation, three separate methods were utilized for congeners of the CTX4A-group, CTX3C-group, and congeners of C- and I-CTX-group, respectively.

The following parameters were applied to all congeners and product ions: ion spray voltage 5500 V; source temperature 250 °C; gas 1 and 2, each 60 psi; curtain gas 40 psi; DP 60 V; EP 8 V; CXP 10 V; CE 30 eV; and source position (horizontal/vertical) 5.0/5.0.

#### 4.3.3. Analysis of Ammonium Adducts [M + NH_4_]^+^—High-Resolution

An Agilent 1260 Infinity II HPLC (Agilent, Waldbronn, Germany) coupled to a Sciex TripleTOF 6600+ (Sciex, Darmstadt, Germany) was operated for analysis. Data acquisition and data evaluation were performed using the software Analyst TF 1.8.1 and SciexOS-Q, respectively. The separation started with 78% B, and the proportion of B was increased to 92% within 10 min. For column washing, the ratio was increased to 99% within 0.1 min. This setting was held for 3.4 min. Then, the proportion of B% was set back to initial conditions within 0.1 min, and the system was allowed to equilibrate for 4.4 min resulting in a total run-time of 18 min. Flow rate and injection volume were set to 0.55 mL min^−1^ and 5 µL, respectively.

MS analyses were performed as Product Ion (PI) scans in High Sensitivity mode. Ammonium adducts [M + NH_4_]^+^ were selected as precursors. Due to the low resolution of the quadrupole, congeners with similar *m*/*z* for the ammonium adducts were combined in one experiment. The scan range of all PI scans was set to 100–1400 *m*/*z* with an accumulation time of 40 ms. In addition, a full scan was recorded using a scan range of 800–1400 *m*/*z* and an accumulation time of 60 ms.

For evaluation, ion traces of the product ions [M + H]^+^, [M + H − H_2_O]^+^, [M + H − 2H_2_O]^+^, and [M + H − 3H_2_O]^+^ were extracted from the PI scan with an extraction window ±25 mDa around the exact mass (Table 1).

The following parameters were applied to all experiments: ion spray voltage 5500 V, source temperature 400 °C, gas 1 60 psi, gas 2 50 psi, curtain gas 35 psi, DP 70 V, CE 30 eV (PI) or 10 eV (Full Scan), and source position (horizontal/vertical) 5.0/1.0.

### 4.4. Method Validation

In the following, the term “fillet sample” refers to raw wet fish tissue (fillet portion) samples, where a sample weight of (5.00 ± 0.01) g was used for extraction. The term “freeze-dried sample” refers to freeze-dried material with a sample weight of (0.50 ± 0.01) g used for sample preparation. Method validation was performed using four CTXs, namely CTX1B, 52-*epi*-54-deoxyCTX1B, 54-deoxyCTX1B, and CTX3C. Analyses of sodium adducts included all CTX congeners listed in Table 1, if not stated otherwise.

#### 4.4.1. Recovery Rates, Matrix Effects, Extraction Efficiency, and Sample Stability

Recovery rates were determined using three fish species (*L. malabaricus*, *S. ghobban*, and *E. aerolatus*). For each species (*n* = 3), fillet and freeze-dried samples (*n* = 2) were investigated, resulting in six different matrices tested in total. Each sample was fortified with 125 µL of a mixed standard solution containing 8 µg L^−1^ for CTX1B, 52-*epi*-54-deoxyCTX1B, 54-deoxyCTX1B, and 16 µg L^−1^ for CTX3C, corresponding to a content of 0.2/0.4 µg kg^−1^ (ppb) for fillet and 2/4 ppb for freeze-dried samples, respectively. The fortified samples were incubated at room temperature for 10 min to allow interaction of the standards with the matrix surface, followed by the sample preparation procedure outlined in Section 4.2.

The samples were analyzed on the day of preparation (*t* = 0) via LC-MS/MS (Section 4.3.1) performing the triplicate injection. Recovery rates were calculated based on a single-point calibration using a standard with 1.33/2.67 µg L^−1^ (fourfold injection). The standard dilution was prepared from the same mixed standard utilized for sample fortification. Recovery rates of individual injections were calculated according to Equation (1)

(1) $$\mathrm{Recovery}\;\mathrm{rate}\;\left(\mathrm{RR}\right)\;\left[\%\right]=\frac{\mathrm{Area}\;(\mathrm{sample})}{\mathrm{Area}\;(\mathrm{standard})}\times \frac{c\;(\mathrm{standard})}{c\;(\mathrm{sample})}\times 100\%,$$

with c (sample) corresponding to the theoretical concentration in the final extract (2/4 µg L^−1^), assuming recovery of 100% and no matrix effects during LC-MS/MS analysis. For each matrix, the mean of the individual recovery rates (*n* = 3) was used for further evaluation.

On the same working day, blank sample extracts of the six matrices were prepared. Blank extracts were analyzed by LC-MS/MS (Section 4.3.1, Section 4.3.2 and Section 4.3.3) to check for potential matrix peak interferences. Furthermore, blank extracts were utilized for the evaluation of matrix effects in both filtrate and eluate samples. For this, 30 µL extract was mixed with 10 µL mixed standard solution containing 4 µg L^−1^ for CTX1B, 52-*epi*-54-deoxyCTX1B, 54-deoxyCTX1B, and 8 µg L^−1^ for CTX3C. Samples were analyzed on the day of preparation (*t* = 0) via LC-MS/MS (Section 4.3.1). Analysis was performed in triplicate (except day 26 for stability testing, double injection). Matrix effects were calculated based on a single-point calibration using a standard with 1/2 µg L^−1^ (fourfold injection; except day 26, double injection). The standard dilution was prepared from the same mixed standard utilized for extract fortification. Matrix effects of individual injections were calculated according to Equation (2),

(2) $$\mathrm{Matrix}\;\mathrm{effect}\;\left(\mathrm{ME}\right)\;\left[\%\right]=\frac{\mathrm{Area}\;(\mathrm{sample})}{\mathrm{Area}\;(\mathrm{standard})}\times \frac{c\;(\mathrm{standard})}{c\;(\mathrm{sample})}\times 100\%,$$

with c (sample) and c (standard) possessing the same (theoretical) concentration in this case. The mean of matrix effects (*n* = 3, day 26 *n* = 2) was used for further evaluation.

According to [60], the data obtained for recovery rates and matrix effects can be used to calculate the extraction efficiency, i.e., the analyte recovery from the matrix without matrix effects during analysis (Equation (3)). The means of both parameters were utilized for calculation.

(3) $$\mathrm{Extraction}\;\mathrm{efficiency}\;\left(\mathrm{EE}\right)\;\left[\%\right]=\frac{\mathrm{RR}}{\mathrm{ME}}\times 100\%$$

To estimate the analyte stability in the extract, the extracts were reanalyzed after 6, 13, 19, and 26 days. The reference mixed standard was prepared each working day from the stock solution. To investigate any potential changes to the matrix during storage and the potential impact on the recovery rate, matrix effect samples and the corresponding standard dilution were freshly prepared and analyzed on the same day.

#### 4.4.2. Limit of Detection, Limit of Quantitation, and Linearity

Limits of detection (LOD) and quantitation (LOQ), as well as linearity, were determined for matrix extracts (snapper, fillet, and freeze-dried) and solvent standards. All solutions (matrix extracts, solvent standards) were prepared in methanol. LOD and LOQ were determined by the signal-to-noise ratio (S/N) and triplicate injection. LOD and LOQ were defined as S/N ≥ 3 and S/N ≥ 9, respectively (according to [47], S/N between 6 and 10 are utilized for LOQ determination). Linearity was tested according to the Mandel adaptation test [61]. Due to the limited availability of standards, the number of concentration levels was reduced to a minimum. Instead, different injection volumes were utilized, ranging from 1.0 to 2.5 µL for matrix samples, and 1.0 to 5.0 µL in the case of solvent standards. This procedure was used for the linearity test (matrix and solvent standards) and LOD/LOQ determination in the case of solvent standards. Final concentrations were calculated based on an injection volume of 2 µL.

For LOD/LOQ determination in matrix samples, the snapper extracts prepared for recovery rate determination (Section 4.4.1) were diluted with a snapper blank matrix in ratios of 1:2, 1:5, 1:10, and 1:20 (*v*/*v*). The procedure was conducted both for eluate and filtrate samples, as well as fillet and freeze-dried material. This way, the obtained LOD/LOQ include matrix effects and losses during sample preparation. Samples were analyzed using the screening method with 20 transitions (Section 4.3.1). Additionally, the 1:20 dilution was analyzed with a “reduced method” that contained three transitions of the standard compounds only. An injection volume of 2 µL was utilized in all cases.

For linearity analysis, the eluate of blank snapper extracts (fillet and freeze-dried) was fortified with a mixed standard. In order to maintain a constant matrix level over the entire concentration range, a matrix proportion of 50% was utilized in all dilutions. Concentrations up to 5 µg L^−1^ (CTX3C 10 µg L^−1^) were tested. The lower end of the linear working range was defined based on the LOQ determined for standard solutions and the matrix effects determined for the respective analyte in the matrices utilized. For solvent standards, linearity was tested up to 10 µg L^−1^ (CTX3C 20 µg L^−1^).

#### 4.4.3. Method Precision—Repeatability

For determining the repeatability [62], naturally contaminated snapper samples (*L. bohar*) were utilized. The sample material was obtained during a CP incident in Germany in 2017 [58]. Aliquots of several fillet samples were combined, freeze-dried, and homogenized (see Section 4.1). Four samples were prepared (Section 4.2) and analyzed (Section 4.3.1), performing a duplicate injection per sample. Repeatability was calculated as relative standard deviation (%RSD) based on peak areas of several potential CTX congeners that were selected based on screening as well as low- and high-resolution confirmation analyses (Section 4.3.2 and Section 4.3.3, detailed discussion for peak selection in Section 2.3).

### 4.5. Extract Performance in the N2a-Assay

The extracts, prepared according to Section 4.2, were evaluated by the N2a-MTT-assay (MTT: 3-(4,5-dimethylthiazol-2-yl)-2,5-diphenyltetrazolium bromide) using Mouse (*Mus musculus*) neuroblastoma type cells, from the cell line Neuro-2a (ATCC^®^ CCL-131™). Cells were purchased from the American Type Culture Collection (LGC Standards GmbH Wesel, Germany) from the lot Numbered 63649750, which was frozen 24 February 2016 at passage number 184. Cell line maintenance and dosing procedures are described in [22,63,64], with cell line modifications described in [59]. For each sample, an eight-point dose-response curve was performed, and cellular responses were compared among samples.

Blank matrix extracts of snapper (fillet and freeze-dried) were prepared and tested via the N2a-assay to investigate any potential matrix interferences (i.e., growth or non-specific cell death), based on the starting tissue type extract, and evaluated for any interfering matrix effects on cell performance when compared to non-exposed control cells. The tissue equivalent (TE) concentration range applied was between 0.24–31.25 mg wet TE (i.e., fillet) and 0.024–3.13 mg dry TE (i.e., freeze-dried), respectively. Eluate and filtrate samples were utilized in combination and independently in order to evaluate the individual contributions or potential impacts on the cell assay.

Additionally, naturally contaminated snapper samples (*L. bohar*) were investigated. The sample material was obtained during a CP incident in Germany in 2017 [58]. Three independent fillet samples were extracted, representing low, medium, and high toxicity. Eluate and filtrate portions of the extracts were evaluated separately and in combination. The range of mg TE applied among all samples was between 0.005 and 30 mg wet TE per well.

The same sample material was extracted using a protocol described by [22], with slight modifications. Briefly, (5.00 ± 0.01) g fish fillets were homogenized by ultra turrax and subsequently extracted twice, with 15 and 10 mL acetone. The extract was evaporated to dryness in a stream of nitrogen at 40 °C, and the residue was reconstituted in 5 mL 80 vol% methanol. Defatting was performed twice with 5 mL *n*-hexane each. The methanol phase was reduced to dryness. After adding 5 mL water, CTXs were extracted twice with chloroform (two times 5 mL). The extract was reduced to dryness and reconstituted in 50 µL chloroform. The sample was applied to a Bond Elut SI cartridge, conditioned with 5 vol% water in methanol, 100% methanol, and chloroform. The sample vessel was rinsed three times with 200 µL chloroform, and the combined rinse solvent was applied to the column. The cartridge was washed with one column volume of chloroform. Elution was performed with two column volumes of 10 vol% methanol in chloroform. The eluate was reduced to dryness and reconstituted in 1 mL methanol and stored in a glass vial at −20 °C until usage. The additional SPE step described by [22] using an amino phase was not conducted as the purity of extracts was sufficient for N2a-assay.

## Figures and Tables

**Table 2:** Matrix effects (ME), recovery rates (RR), and extraction efficiencies (EE) of fortified CTX in different matrices.

Day	0	6	13	19	26	0	6	13	19	26	0	6	13	19	26
	ME					RR					EE				
**CTX1B**															
snapper, fillet	75	75	69	86	85	47	43	40	47	46	64	57	58	54	54
snapper, freeze-dried	76	67	71	81	83	47	40	44	48	45	62	60	61	60	54
parrotfish, fillet	92	85	89	100	97	62	62	55	62	55	68	72	62	61	57
parrotfish, freeze-dried	90	86	84	95	97	57	56	54	62	53	63	65	65	65	54
grouper, fillet	66	54	61	77	62	39	36	36	41	34	59	66	59	53	56
grouper, freeze-dried	74	67	66	82	82	45	41	40	50	48	61	61	61	60	59
**52*-epi-*54-deoxyCTX1B**															
snapper, fillet	100	96	81	99	88	63	53	47	61	46	63	55	59	61	52
snapper, freeze-dried	84	78	78	95	83	57	53	54	60	53	67	67	69	64	63
parrotfish, fillet	93	94	93	98	96	66	59	62	69	59	72	63	67	71	62
parrotfish, freeze-dried	103	103	95	97	104	71	65	64	77	65	69	63	67	80	62
grouper, fillet	70	60	57	70	62	35	33	33	43	35	50	55	57	61	56
grouper, freeze-dried	66	69	66	85	75	52	43	38	55	44	79	62	58	65	59
**54-deoxyCTX1B**															
snapper, fillet	73	73	69	85	82	42	43	40	47	46	58	59	59	55	57
snapper, freeze-dried	79	68	65	84	88	48	45	47	55	49	61	66	71	66	56
parrotfish, fillet	90	83	84	97	104	64	64	58	67	58	71	77	68	69	56
parrotfish, freeze-dried	93	87	89	96	101	69	66	61	72	64	84	76	68	75	63
grouper, fillet	66	63	62	76	62	35	36	37	43	39	53	58	60	56	63
grouper, freeze-dried	72	73	72	86	81	48	47	45	55	52	66	65	62	64	64
**CTX3C—filtrate**															
snapper, fillet	95	94	87	110	102	36	38	34	38	41	38	41	39	35	40
snapper, freeze-dried	98	102	100	121	114	40	41	41	46	49	41	40	41	38	43
parrotfish, fillet	116	112	102	101	130	48	47	41	52	52	42	42	41	52	40
parrotfish, freeze-dried	109	118	107	112	128	53	56	50	61	61	49	47	46	55	47
grouper, fillet	85	90	84	100	98	40	38	35	40	42	47	42	42	40	43
grouper, freeze-dried	98	113	100	107	125	45	42	41	49	49	46	37	41	46	39
**CTX3C—eluate**															
snapper, fillet	94	91	83	100	82	25	25	24	27	21	26	27	29	27	26
snapper, freeze-dried	95	88	88	100	93	28	28	26	27	24	30	32	30	27	26
parrotfish, fillet	104	102	101	107	97	35	32	29	31	27	33	31	28	29	28
parrotfish, freeze-dried	99	101	96	105	93	35	34	31	32	26	35	33	32	31	28
grouper, fillet	72	61	65	79	63	25	21	23	22	20	34	35	36	28	32
grouper, freeze-dried	89	79	81	97	75	30	27	28	31	29	34	34	34	32	39
**CTX3C—sum**															
snapper, fillet						60	63	58	65	62	64	68	68	62	66
snapper, freeze-dried						68	69	68	73	73	71	72	71	65	69
parrotfish, fillet						83	79	70	83	80	75	73	69	81	68
parrotfish, freeze-dried						88	89	81	93	86	84	81	79	85	75
grouper, fillet						65	59	58	62	62	81	76	78	67	74
grouper, freeze-dried						76	69	69	80	79	80	71	75	78	78
min compared to *t* = 0 *		82	81	87	84		83	74	89	73		79	74	81	74
max compared to *t* = 0 *		115	102	130	127		107	106	123	123		113	117	124	120

All values given in %; values provided as average (*n* = 3; except ME on day 26, here *n* = 2); EE—extraction efficiency, ME—matrix effect, RR—recovery rate, min—minimum, max—maximum; * calculation performed for mean values; information about standard deviation of ME and RR is provided in Table A1.

**Table 3:** Limits of detection and quantitation and linearity ranges for CTX standards in methanol solutions and matrix samples.

Parameter	CTX1B	52*-epi-*54-deoxyCTX1B	54-deoxyCTX1B	CTX3C	
linearity ranges, methanol [µg L^−1^]	0.075–10	0.2–10	0.1–7	0.2–20	
linearity ranges, matrix ^1^ [µg L^−1^]	0.1–5	0.2–5	0.2–5	0.2–10	
LOD methanol [µg L^−1^]	0.02	0.05	0.03	0.06	
LOQ methanol [µg L^−1^]	0.075	0.2	0.1	0.2	
				**CTX3C eluate**	**CTX3C filtrate**
LOD fillet [µg kg^−1^], full ^2^	0.02	0.02	0.02	0.08	0.04
LOD fillet [µg kg^−1^], reduced ^3^	0.01	0.01	0.01	(0.04)	0.02
LOQ fillet [µg kg^−1^], full ^2^	0.04	0.1	0.04	0.4	0.2
LOQ fillet [µg kg^−1^], reduced ^3^	(0.02)	(0.05)	(0.02)	(0.2)	(0.1)
LOD freeze-dried [µg kg^−1^], full ^2^	0.2	0.2	0.1	0.4	0.2
LOD freeze-dried [µg kg^−1^], reduced ^3^	0.1	0.1	(0.05)	(0.2)	0.2
LOQ freeze-dried [µg kg^−1^], full ^2^	0.4	1	0.4	0.8	0.8
LOQ freeze-dried [µg kg^−1^], reduced ^3^	(0.2)	(0.5)	0.1	(0.4)	(0.4)

^1^ determined for snapper eluates, both fillet and freeze-dried; same values for both matrices, ^2^ full method with 20 transitions recorded, ^3^ reduced method with three transitions recorded (see Section 4.4.2 for details); values in brackets (##) were calculated based on factor 2 (observed sensitivity difference between full and reduced method); LOD—limit of detection, LOQ—limit of quantitation.

**Table 4:** Retention times of P-CTX congeners reported in the literature compared to the standard compounds used in this study.

Congener	[14]	[49]	This Study ^1^	This Study ^2^
2,3,51-trihydroxyCTX3C	4.52			
4-hydroxy-7-oxoCTX1B	4.58			
7-oxoCTX1B	4.71			
7-hydroxyCTX1B	4.71			
A-*seco*-51-hydroxyCTX3C	4.75			
CTX1B	4.95	2.6	3.1	3.0
M-*seco*-CTX3C	6.21	4.7		
2,3-dihydroxyCTX3C	7.80	5.9		
51-hydroxyCTX3C	7.24	6.1		
M-*seco*-CTX4A/B	7.19	6.6		
52*-epi-*54-deoxyCTX1B	7.87	6.7	5.9	6.2
51-hydroxy-2-oxoCTX3C	8.15			
54-deoxyCTX1B	8.30	7.2	6.2	6.6
2-hydroxyCTX3C	8.94	7.3		
M-*seco*-CTX3C methyl acetal		10.5		
CTX3B	14.50	11.5		
CTX3C	15.44	11.8	9.4	10.0
CTX4A	15.30	12.9		
CTX4B		13.3		

Retention times given in minutes; ^1^ setup described in Section 4.3.1 and Section 4.3.2; ^2^ setup described in Section 4.3.3.

**Table 5:** Retention times of C- and I-CTX congeners reported in the literature compared to the standard compounds used in this study.

Congener	[16]	[8]	[9]	[50] ^1^	[50] ^2^	This Study ^3^	This Study ^4^
C-CTX-3/4	3.70/4.11						
C-CTX-1/2	6.4		62	4.6	9.4		
C-CTX reaction product 9	8.0						
C-CTX reaction product 8	9.82						
C-CTX-1127					10.6		
C-CTX-1157					7.6		
I-CTX-3/4		5.49/5.60					
I-CTX-6		5.82					
I-CTX-1/2		6.49/6.60	62.5				
I-CTX-5		6.95					
CTX1B			59	3.7		3.1	3.0
52*-epi-*54-deoxyCTX1B				7.4		5.9	6.2
54-deoxyCTX1B				7.8		6.2	6.6

Retention times given in minutes; ^1^ analytical method; ^2^ preparative method; ^3^ setup described in Section 4.3.1 and Section 4.3.2; ^4^ setup described in Section 4.3.3.

## Data Availability

The data presented in this study are available on request from the corresponding author.

## Appendix A

**Table A1:** Matrix effects (ME) and recovery rates (RR) of fortified CTX in different matrices.

Day	0	6	13	19	26	0	6	13	19	26
	ME					RR				
**CTX1B**										
snapper, fillet	75 ± 4	75 ± 2	69 ± 3	86 ± 9	85 ± 4	47 ± 2	43 ± 1	40 ± 6	47 ± 5	46 ± 2
snapper, freeze-dried	76 ± 3	67 ± 2	71 ± 4	81 ± 7	83 ± 1	47 ± 1	40 ± 0	44 ± 5	48 ± 3	45 ± 4
parrotfish, fillet	92 ± 4	85 ± 3	89 ± 7	100 ± 3	97 ± 3	62 ± 6	62 ± 2	55 ± 5	62 ± 3	55 ± 3
parrotfish, freeze-dried	90 ± 1	86 ± 4	84 ± 5	95 ± 6	97 ± 10	57 ± 5	56 ± 4	54 ± 3	62 ± 3	53 ± 6
grouper, fillet	66 ± 5	54 ± 3	61 ± 1	77 ± 2	62 ± 1	39 ± 2	36 ± 2	36 ± 2	41 ± 0	34 ± 2
grouper, freeze-dried	74 ± 4	67 ± 4	66 ± 5	82 ± 5	82 ± 8	45 ± 2	41 ± 3	40 ± 2	50 ± 3	48 ± 1
**52*-epi-*54-deoxyCTX1B**										
snapper, fillet	100 ± 5	96 ± 2	81 ± 11	99 ± 6	88 ± 10	63 ± 1	53 ± 4	47 ± 6	61 ± 5	46 ± 3
snapper, freeze-dried	84 ± 1	78 ± 4	78 ± 2	95 ± 1	83 ± 6	57 ± 3	53 ± 4	54 ± 5	60 ± 1	53 ± 4
parrotfish, fillet	93 ± 6	94 ± 5	93 ± 7	98 ± 4	96 ± 2	66 ± 3	59 ± 0	62 ± 9	69 ± 2	59 ± 3
parrotfish, freeze-dried	103 ± 4	103 ± 2	95 ± 2	97 ± 10	104 ± 3	71 ± 2	65 ± 1	64 ± 5	77 ± 2	65 ± 8
grouper, fillet	70 ± 2	60 ± 7	57 ± 0	70 ± 2	62 ± 5	35 ± 2	33 ± 1	33 ± 2	43 ± 2	35 ± 2
grouper, freeze-dried	66 ± 7	69 ± 1	66 ± 10	85 ± 6	75 ± 4	52 ± 2	43 ± 3	38 ± 1	55 ± 3	44 ± 2
**54-deoxyCTX1B**										
snapper, fillet	73 ± 2	73 ± 1	69 ± 2	85 ± 6	82 ± 1	42 ± 2	43 ± 1	40 ± 2	47 ± 0	46 ± 3
snapper, freeze-dried	79 ± 6	68 ± 2	65 ± 1	84 ± 5	88 ± 3	48 ± 3	45 ± 2	47 ± 5	55 ± 2	49 ± 1
parrotfish, fillet	90 ± 1	83 ± 2	84 ± 2	97 ± 7	104 ± 3	64 ± 6	64 ± 1	58 ± 1	67 ± 1	58 ± 5
parrotfish, freeze-dried	93 ± 3	87 ± 1	89 ± 2	96 ± 7	101 ± 1	69 ± 2	66 ± 1	61 ± 7	72 ± 2	64 ± 4
grouper, fillet	66 ± 3	63 ± 2	62 ± 4	76 ± 2	62 ± 13	35 ± 1	36 ± 2	37 ± 3	43 ± 1	39 ± 3
grouper, freeze-dried	72 ± 5	73 ± 2	72 ± 5	86 ± 1	81 ± 7	48 ± 2	47 ± 1	45 ± 3	55 ± 3	52 ± 3
**CTX3C—filtrate**										
snapper, fillet	95 ± 6	94 ± 3	87 ± 12	110 ± 6	102 ± 3	36 ± 6	38 ± 4	34 ± 2	38 ± 1	41 ± 2
snapper, freeze-dried	98 ± 1	102 ± 2	100 ± 11	121 ± 17	114 ± 3	40 ± 4	41 ± 1	41 ± 0	46 ± 1	49 ± 3
parrotfish, fillet	116 ± 5	112 ± 7	102 ± 6	101 ± 4	130 ± 11	48 ± 5	47 ± 1	41 ± 2	52 ± 1	52 ± 6
parrotfish, freeze-dried	109 ± 7	118 ± 10	107 ± 6	112 ± 13	128 ± 11	53 ± 6	56 ± 1	50 ± 3	61 ± 4	61 ± 2
grouper, fillet	85 ± 6	90 ± 4	84 ± 3	100 ± 10	98 ± 4	40 ± 3	38 ± 3	35 ± 2	40 ± 2	42 ± 2
grouper, freeze-dried	98 ± 4	113 ± 4	100 ± 4	107 ± 10	125 ± 10	45 ± 5	42 ± 7	41 ± 3	49 ± 3	49 ± 2
**CTX3C—eluate**										
snapper, fillet	94 ± 3	91 ± 4	83 ± 9	100 ± 11	82 ± 7	25 ± 0	25 ± 3	24 ± 3	27 ± 2	21 ± 6
snapper, freeze-dried	95 ± 9	88 ± 5	88 ± 4	100 ± 4	93 ± 4	28 ± 2	28 ± 1	26 ± 3	27 ± 1	24 ± 3
parrotfish, fillet	104 ± 4	102 ± 5	101 ± 3	107 ± 5	97 ± 17	35 ± 1	32 ± 2	29 ± 3	31 ± 1	27 ± 3
parrotfish, freeze-dried	99 ± 3	101 ± 5	96 ± 8	105 ± 7	93 ± 7	35 ± 2	34 ± 1	31 ± 6	32 ± 0	26 ± 6
grouper, fillet	72 ± 5	61 ± 4	65 ± 5	79 ± 6	63 ± 1	25 ± 1	21 ± 1	23 ± 1	22 ± 3	20 ± 1
grouper, freeze-dried	89 ± 3	79 ± 2	81 ± 5	97 ± 2	75 ± 10	30 ± 1	27 ± 1	28 ± 4	31 ± 1	29 ± 7

All values given in %; values provided as average (*n* = 3; except ME on day 26, here *n* = 2) ± standard deviation; EE—extraction efficiency, ME—matrix effect.
